# Structural design and safety performance of a novel high-strength steel lightweight guardrail

**DOI:** 10.1371/journal.pone.0317353

**Published:** 2025-01-24

**Authors:** Hongliang Wei, Yongke Wei, Zhenhua Dai, Tingquan He, Changsong Wu, Dongmin Peng, Feng Zhang

**Affiliations:** 1 Guangxi Zhuang Autonomous Region Expressway Development Center, Nanning, China; 2 Guangxi XinFaZhan Communication Group Co., LTD, Nanning, China; 3 Key Laboratory of Road and Traffic Engineering of Ministry of Education, Tongji University, Shanghai, China; 4 Jiangsu Guoqiang New Material Technology LTD, Changzhou, China; Islamic Azad University Mashhad Branch, ISLAMIC REPUBLIC OF IRAN

## Abstract

Highway guardrails are critical safety infrastructure along roadways, designed to redirect vehicles back into their lanes and facilitate a gradual deceleration to a complete stop. Traditional highway steel guardrails exhibit significant limitations, including inadequate energy absorption, susceptibility to corrosion, and an increased risk of vehicles leaving the roadway during severe collisions. Furthermore, the production and transportation of these guardrails contribute to substantial carbon emissions and environmental pollution. This study presents an optimization of the cross-sectional shape of the conventional corrugated beam guardrail, proposing a lightweight structure that incorporates high yield strength steel plate HR700F to enhance energy absorption capacity. The safety performance of the proposed guardrail is rigorously assessed through finite element numerical simulations and full-scale collision tests with real vehicles. Key performance indicators—such as the maximum dynamic lateral deflection of the guardrail, occupant impact velocity, and acceleration—are utilized to evaluate the energy absorption and protective efficacy of the structure. Results indicate that the optimized guardrail not only meets SB-level safety standards but also demonstrates superior anti-collision performance and effective energy absorption and buffering characteristics. The proposed design achieves a reduction in beam plate thickness by 1.3 mm, resulting in a lightweight structure with a weight reduction of up to 44%, thereby supporting the advancement of low-carbon, environmentally sustainable transportation solutions.

## 1. Introduction

Corrugated steel guardrails are the most widely utilized road traffic safety protection facilities [1], playing a crucial role in safely controlling and redirecting out-of-control vehicles [2]. These structures effectively prevent vehicles from running off the road or crossing the central divider into opposing lanes, thereby significantly mitigating the severity of traffic accidents [3]. As of 2023, China’s total highway mileage is projected to reach 5.4368 million kilometers, with annual steel consumption for guardrails exceeding 5 million tons, highlighting the substantial demand for these safety installations.

The predominant material used for corrugated steel guardrails is Q235 low-carbon steel, which presents several drawbacks, including low material strength, rapid corrosion in soil environments [4], and a relatively high unit volume weight. The typical service life of these guardrails is often less than the intended lifespan of 15 years. Moreover, galvanized guardrail plates incur high steel consumption and production costs, while the hot-dip galvanizing process poses significant environmental pollution challenges [5]. Carbon emissions during the production and transportation of guardrails are considerable. For instance, a 4mm three-corrugated steel guardrail weighs approximately 102 kg and requires at least four workers for installation, complicating replacement in the event of damage and resulting in a low material reuse rate. In coastal regions, these guardrails are particularly susceptible to rust, further diminishing their service life. Road traffic carbon emissions account for roughly 74% of total emissions in the transportation sector, positioning it as the second largest contributor to greenhouse gas emissions [6, 7]. The emerging emphasis on green and low-carbon development necessitates new sustainability standards for traffic safety facilities. Transitioning to environmentally friendly highway guardrails is a vital component of the low-carbon transformation within the transportation sector.

Currently, both domestic and international research teams have conducted extensive studies on the structural and material design of highway guardrails. Yin et al. [8]. developed a novel eta-shaped W-beam guardrail (eta-WG) aimed at preventing tire jamming and enhancing the redirection function of guardrails. Jiang et al. [9]. introduced a new type of anti-collision guardrail constructed from recycled foam concrete within a thin-walled steel shell, assessing its failure process through impact tests and numerical simulations. Yang [10] proposed a movable intermediate guardrail that addresses the structural deficiencies of traditional designs, thereby improving collision performance. Zhao et al. [11] investigated the mechanism of column stumbling, designing filled columns and N-shaped curved columns. Their orthogonal tests revealed that these modifications can significantly reduce column stumbling and enhance the collision safety of the guardrail. Wang et al. [12] utilized the high ductility of aluminum alloy (Al) to enhance the cross-sectional shape of guardrails, demonstrating that this new design effectively minimizes deformation and lowers the risk of vehicles leaving the roadway. Zhang et al. [13] designed a movable steel guardrail featuring a lightweight composite corrugated beam, which was evaluated for safety through finite element simulation and real vehicle collision tests. Li et al. [14] developed a double-layer guardrail that combines rigidity and flexibility. Safety performance evaluations indicated that this design effectively reduces harm to drivers and passengers during collision events, providing a more significant buffering effect. These advancements in guardrail design underscore the ongoing efforts to improve safety and sustainability in traffic management systems.

In summary, existing research has primarily addressed the shortcomings of traditional steel materials through advancements in structural design, installation layout, and material selection, yielding notable results. However, studies focusing on lightweight structures and the environmental benefits of guardrails with superior energy absorption capacity remain limited. This study aims to elucidate the functional characteristics of high-strength steel lightweight guardrails concerning anti-collision performance, buffering capabilities, and energy absorption. For this purpose, HR700F high-strength steel was selected due to its attributes of high strength, high elongation, and exceptional corrosion resistance. Various guardrail structures, forms, and materials were examined, and different comparison groups were established to optimize the guardrail design. The proposed design features a new type of corrugated beam guardrail that minimizes material usage while maintaining safety. To assess whether the new guardrail design meets the safety performance standards for real traffic collision scenarios, we employed finite element simulation followed by real vehicle collision tests. These evaluations were conducted to ensure compliance with the anti-collision level requirements specified in relevant standards. The results indicate that high-strength steel lightweight guardrails effectively reduce the weight of materials while enhancing the durability of the facilities, thereby achieving significant energy savings and cost reductions.

## 2. Structure design and evaluation standard of guardrail

### 2.1. High-strength lightweight guardrail structure

The corrugated beam guardrail primarily relies on the deformation of its structure to absorb the energy of colliding vehicles, thereby providing buffering, guidance, and protection for vehicles and drivers. The inherent corrugated design is known for its efficient energy absorption capabilities, making the development of more effective and reliable lightweight energy-absorbing structures a promising area of research [15]. This study utilizes high-performance steel (HHS) HR700F [16], which conforms to the Chinese national standard GB/T20887 and is equivalent to international standards such as FeE700 and the European standard S700MC.HR700F exhibits a tensile strength of up to 750 MPa, a yield strength exceeding 700 MPa, and an elongation of 20%. These mechanical properties represent an improvement of more than 50% compared to traditional carbon steel, such as Q235, which has a tensile strength of approximately 430 MPa and a yield strength of 275 MPa. Consequently, HR700F is expected to absorb more energy during impact collisions [17]. Despite these advantageous properties, further structural design and comprehensive safety performance testing are necessary to validate the reliability of HR700F in the context of highway guardrail protection.

The corrugated beam guardrail is typically composed of three main components: a crossbeam, a column, and a connector. During the collision process, both the column and the foundation soil (or anchor bolt) collectively bear the impact force of the vehicle. They absorb most of the energy through the coordinated deformation of the guardrail structure itself and the interaction with the foundation soil or ground connector [18]. The energy absorption characteristics of the guardrail structure primarily depend on the bending deformation of the crossbeam and the plastic bending deformation of the column. Currently, the design and construction of metal beam-column guardrails rely on established practices, with no definitive guidelines provided for optimal column and crossbeam dimensions. To address the shortcomings of traditional semi-rigid steel guardrails during collision events, this study aims to explore the optimal wall thickness combinations for the guardrail column and crossbeam. We established simulated collision scenarios for these guardrail combinations using finite element simulations, allowing for a comparative analysis of guardrail deformation under consistent collision conditions. Building on previous research regarding the mechanisms of elastic-plastic beam finite element analysis [12], we assume the guardrail can be modeled as a simply supported beam to describe the collision process, as expressed in the following formula.


$$m\frac{\partial^{2}u}{\partial t^{2}}+EI\frac{\partial^{4}u}{\partial x^{4}}=\partial (t_{0})$$ (1)

Where *m* is the mass per unit length of the corrugated beam, *EI* is the bending stiffness of the corrugated beam, and ∂(*t*_0_) is the Dirac delta function, which represents the impact force received by the specimen during the collision.

**Fig 1:**
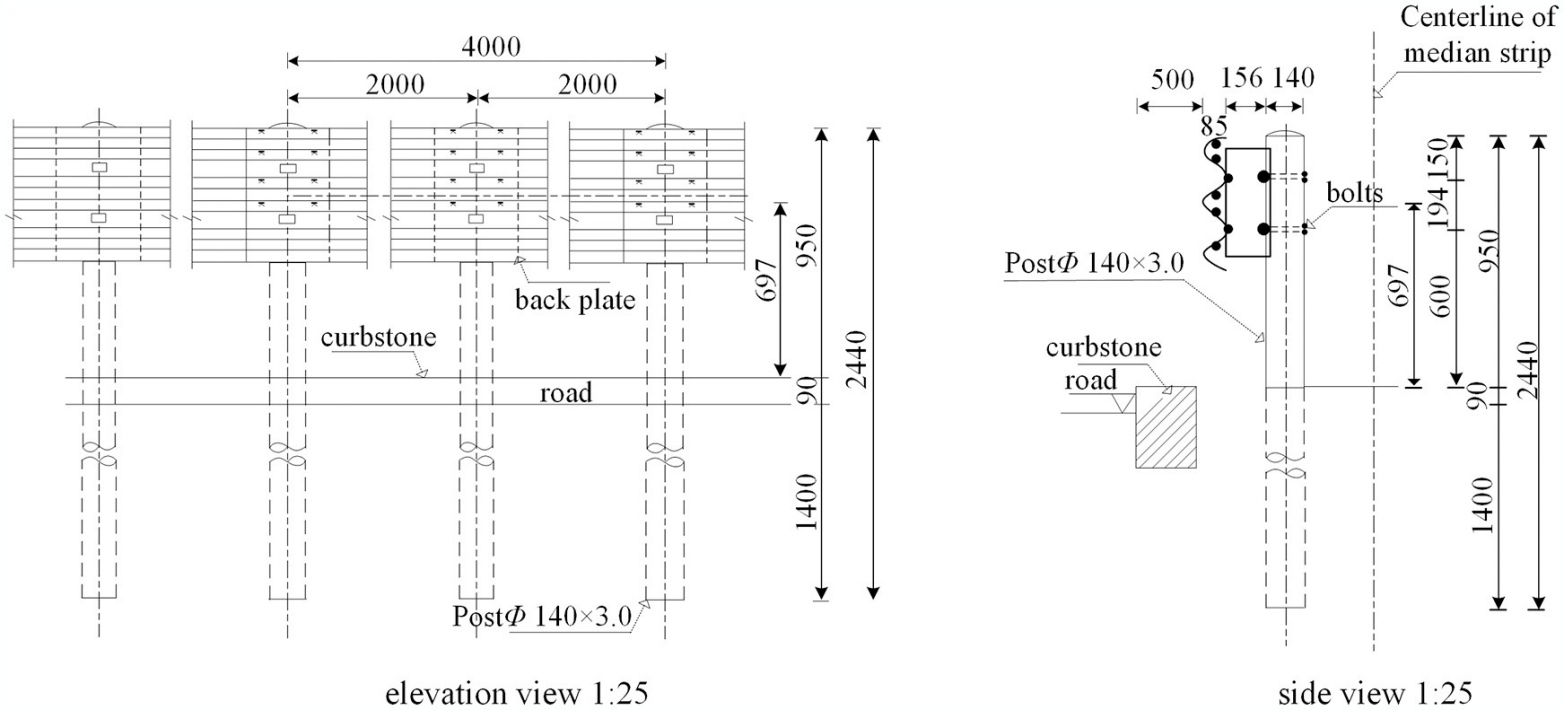
High-strength lightweight guardrail (unit: mm).

Through the application of orthogonal experiments and comprehensive comparisons of various design schemes, we have developed a lightweight structural design for the corrugated beam guardrail that meets the required design protection energy, as illustrated in Fig 1. The optimization process involved adjustments to the thickness of the corrugated plate, the dimensions of the anti-blocking block, and the structural configuration of the SB-level column associated with the new corrugated beam steel guardrail. Post-optimization, the specifications are as follows: the thickness of the corrugated plate is set at 2.7 mm, the wall thickness of the round column is 3.0 mm, the column length is 2440 mm, and the thickness of the anti-blocking block is also 3.0 mm. When compared to conventional carbon structural steel guardrails of similar grade, the steel consumption of the proposed design has decreased significantly, from 61.86 tons/km to 34.17 tons/km, resulting in a substantial 44% reduction in steel usage.

### 2.2. Evaluation criteria of guardrail performance

**Fig 2:**
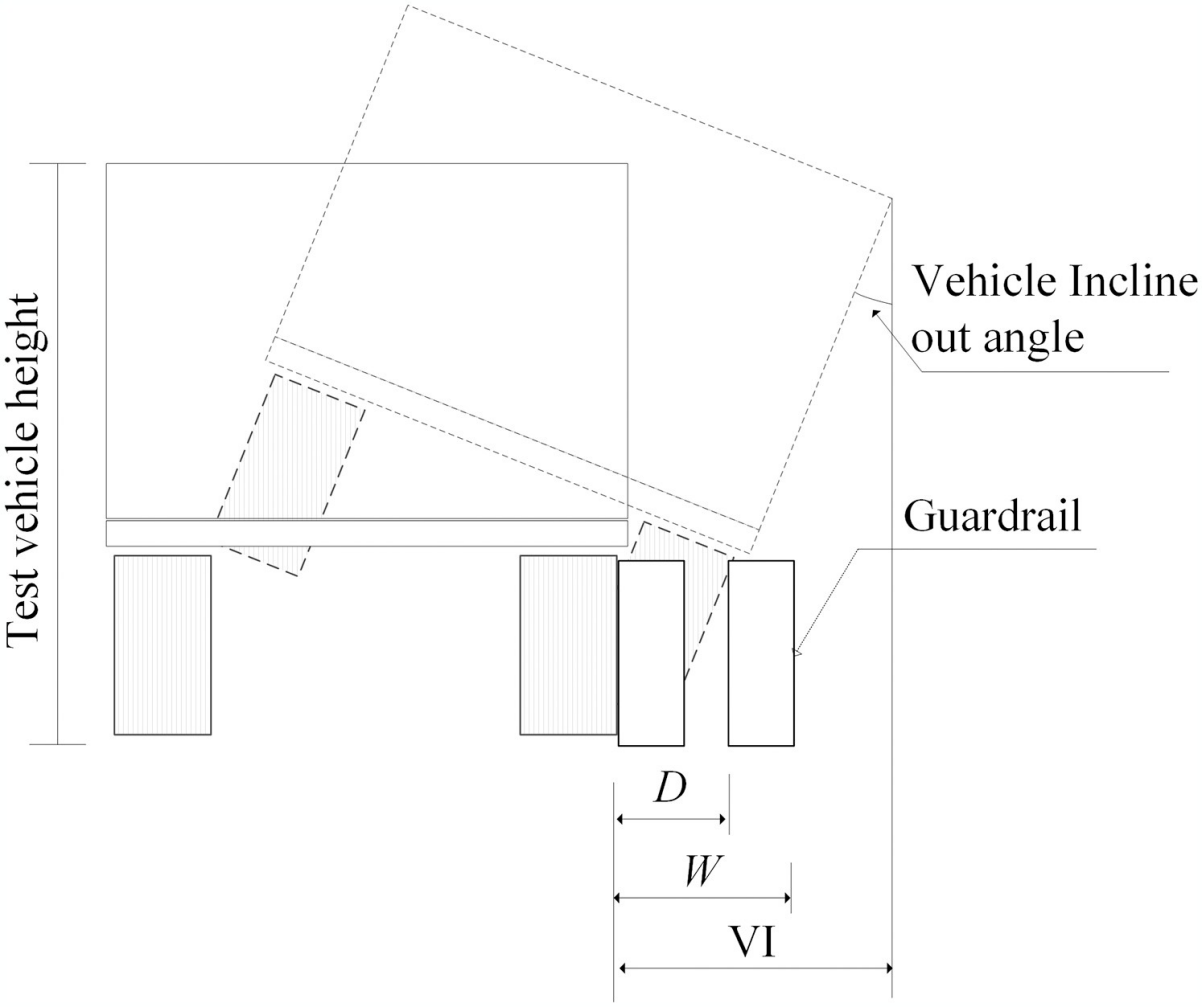
Terminology of vehicle impact with guardrail in SSPEB.

The primary evaluation indicators for the safety performance of highway guardrails include containment performance, buffering performance, and re-directive performance. This evaluation is conducted in accordance with the requirements outlined in the JTG B05-01 2013 Standard for Safety Performance Evaluation of Highway Barriers (SSPEB) [19]. Fig 2 shows the key indicator terms that characterize the collision process. D represents the maximum dynamic lateral deflection of highway guardrail after impact, W represents the maximum dynamic widening distance of lateral deflection of highway guardrail, and VI represents the maximum dynamic vehicle incline-out distance. According to the standard, guardrails are categorized into eight levels based on their protective capabilities. The high-strength and lightweight guardrail developed in this study is classified as level 4 (SB). Table 1 outlines the collision test conditions applicable to level SB, providing a framework for assessing the effectiveness of the proposed guardrail design.

**Table 1:** SB-level collision conditions.

Protection level	Collision vehicle type	Collision velocity/(km·h^-1^)	Collision angle/°	Gross Vehicle Mass/t
SB Level	Small car	100	20	1.5
	Medium bus	80	20	10
	Heavy truck	60	20	18

The blocking function of a highway guardrail is crucial in preventing vehicles from crossing, climbing over, or riding over the guardrail. To assess the buffering function of highway guardrails, two key indicators are utilized: occupant impact velocity (OIV) and occupant ridedown acceleration (ORA). The specific evaluation criteria are established as follows: The longitudinal and lateral components of the occupant impact velocity must remain below 12 m/s. The longitudinal and lateral components of the occupant ridedown acceleration should not exceed 200 m/s^2^.

**Fig 3:**
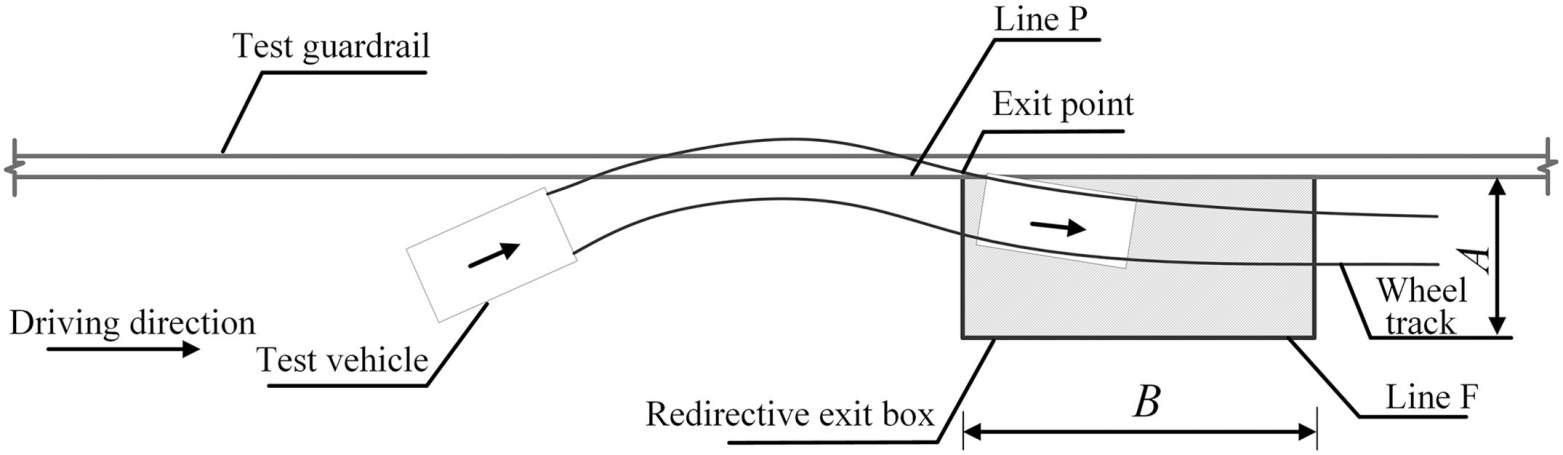
Schematic diagram of collision redirective exit box in SSPEB.

To evaluate the redirective function of highway guardrails, a redirective exit box is introduced, as depicted in Fig 3. The criteria for this evaluation include the following: The vehicle must not overturn upon colliding with the guardrail. The driving trajectory of the vehicle after passing the exit point must remain within the confines of the redirective exit box shown in the figure, ensuring that the vehicle does not cross the straight line F. The parameter *A*’s value range is determined by the vehicle’s dimensions, specifically its width (*V*_*w*_) and length (*V*_*L*_). Additionally, the value of parameter *B* is set at 10 m for small passenger cars and 20 m for larger vehicles. These criteria collectively ensure the effectiveness of the guardrail in enhancing vehicle safety during collision events.


$$\text{A}=\left\{\begin{array}{cc} 2.2{+\text{V}}_{\text{w}}+0.16{\text{V}}_{\text{L}} & \text{small}\;\text{car}\\ 4.4{+\text{V}}_{\text{w}}+0.16{\text{V}}_{\text{L}} & \text{medium}\;\text{bus}\;\text{and}\;\text{heavy}\;\text{truck} \end{array}\right.$$ (2)

## 3. Finite element simulation and analysis

### 3.1. Finite element modeling

The dynamic process occurring during a collision is highly complex, rendering it exceedingly challenging to comprehensively describe the entire collision event using traditional methods that decouple each structure and analyze forces individually. Finite element software alleviates the complexities associated with calculating and analyzing the model. The procedure involves first modeling the vehicle, then using HyperMesh for the pre-processing of the guardrail, and subsequently importing the K file into LS-DYNA to conduct nonlinear explicit dynamic analysis of the collision between the vehicle and the guardrail.

Initially, the finite element meshing of the collision vehicle is performed based on its primary characteristics to establish a finite element model. A simulation finite element model of the guardrail is then created in accordance with its structure and strength. Collision conditions, including speed, angle, and impact point, are predefined, followed by conducting a real vehicle collision test to validate the safety of the guardrail. The accuracy of the simulation results is directly correlated with the degree of similarity between the model and the actual object in the experimental setup. The contact relationship parameters are defined as follows: for vehicle-guardrail and guardrail-guardrail interactions, Contact-Automatic-Surface-to-Surface; for vehicle-road surface interactions, Contact-Automatic-Node-to-Surface; and for vehicle self-contact, Contact-Automatic- Single-Surface. The relevant parameters of the materials comprising the guardrail and soil foundation finite element simulation model are presented in Tables 2 and 3 below.

**Table 2:** Guardrail model material parameters.

RO (density/t/mm3)	E (elastic modules/MPa)	PR (Poisson’s ratio)	SIGY (yield stress/MPa)	FAIL (failure strain)	C (strain rate /s^-1^)
7.850e-09	210000.0	0.3	700.0	1.0	40.0

**Table 3:** Material parameters of soil foundation mode.

RO (density/t/mm^3^)	G (shear modules/MPa)	PR (Poisson’s ratio)	Pc (tensile fracture strength/MPa)	K (bulk modulus/MPa)
1.874e-09	49.5	0.35	-0.55	134.55

**Fig 4:**
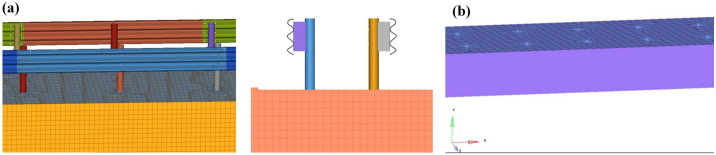
Finite element model construction. (a) Guardrail finite element model; (b) Soil foundation finite element model.

The established finite element model is shown in Fig 4. The core finite element theory of the vehicle-guardrail collision system is large deformation dynamics. The collision model follows the conservation equations. The mass and momentum conservation equations are as follows. Subsequently, the penalty function is used to deal with the collision contact problem and solve the nonlinear motion differential equation.


$$\rho (X,t)J(X,t)=\rho_{0}(X)$$ (3)

$$\frac{\partial \sigma_{ij}}{\partial x}+\rho b_{i}=\rho {\ddot{u}}_{i}$$ (4)

### 3.2. Simulation result

Following the establishment of the vehicle-guardrail finite element model, the nonlinear finite element software LS-DYNA is employed for calculations. LS-DYNA, as a nonlinear finite element solver, is extensively utilized in structural dynamic analysis across various fields, including the military, aerospace, and automotive industries. It is one of the primary tools for conducting automobile safety analyses, capable of simulating a wide array of complex real-world problems and effectively addressing the collision of both two-dimensional and three-dimensional nonlinear structures. Table 4 presents the results of the collision simulation analysis for three types of vehicles. The collision process encompasses typical phases, including initial contact, turning, and parallel movement. Notably, there is no collision between the rear end of the vehicle and the guardrail, nor does the vehicle body experience any reverse turning. Instead, the vehicle continues to move away from the guardrail in its original direction. Throughout the simulation, there is no occurrence of vehicle overturning or straddling the guardrail, and the guardrail remains intact, with no components detaching or fragments invading the vehicle passenger compartment. Additionally, the vehicle’s wheel track remains within the designated guide frame, demonstrating excellent blocking and guiding functions.

**Table 4:** Simulation test of collision safety performance of standard section guardrail.

Type	D/mm	W/mm	VI/mm	VI_n_/mm
Small car	230.0	640.5	-	-
Medium bus	620.5	1042.9	1044.9	1262.0
Heavy truck	622.1	1017.7	932.2	982.1

**Fig 5:**
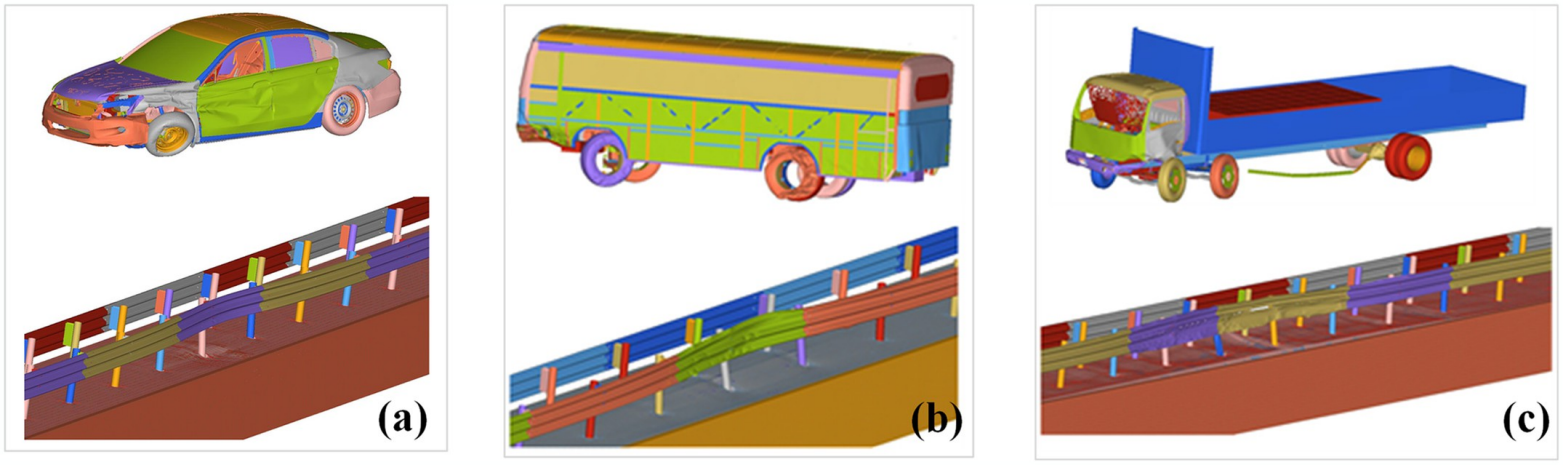
Deformation of vehicle and guardrail. (a)Small car; (b)medium bus; (c) heavy truck.

Fig 5 illustrates the deformation of both the vehicle and guardrail following the simulated collision. Notably, the small passenger car did not penetrate the guardrail, and the occupant collision speed, as well as the acceleration of the occupants in both the *x* and *y* directions, remained within standard limits. In the collision between the medium-sized passenger car and the large truck, both the guardrail deformation and vehicle camber were minimal. Furthermore, the maximum lateral dynamic deformation of the guardrail, the maximum dynamic camber of the vehicle, and the maximum dynamic camber equivalent of the vehicle all complied with the established standard requirements.

## 4. Full-scale collision tests with real vehicles

### 4.1. Experience design

**Fig 6:**
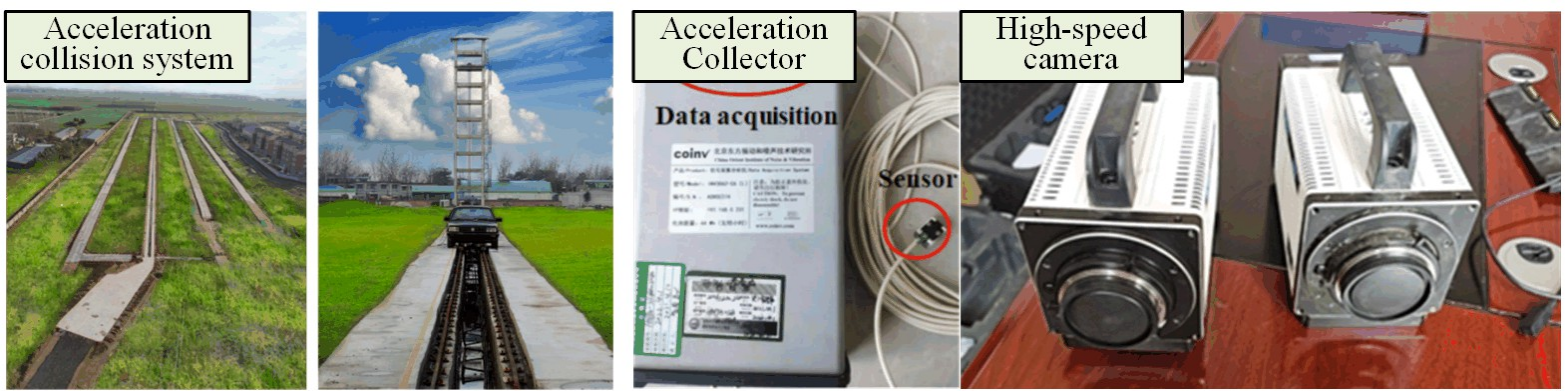
Test site and data acquisition equipment.

The safety performance of highway guardrails should be evaluated through full-scale collision tests involving real vehicles. This testing involves using a full-scale test vehicle to collide with a 1:1 scale guardrail, assessing safety performance based on monitored data. The testing is conducted by a qualified safety facility inspection center. This article outlines the test process in accordance with established standards. Given that the collision between a vehicle and a guardrail is a complex dynamic event, a high-speed camera is employed to capture the vehicle’s trajectory, roll condition, and the deformation process of the guardrail during the impact. Acceleration sensors and data acquisition instruments are installed on the test vehicle to collect collision data, including the lateral and longitudinal accelerations at the center of gravity of the small passenger car. Subsequently, the deformation data of both the vehicle and guardrail before and after the test are compared. The acceleration collision system and instruments utilized in the testing are depicted in Fig 6.

**Fig 7:**
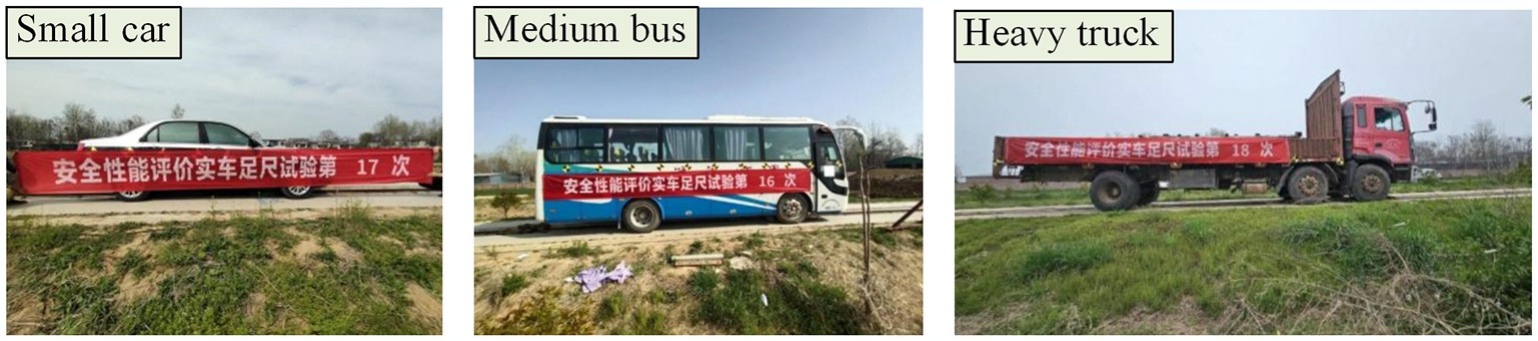
Test vehicles.

Fig 7 displays the test vehicle utilized in the full-scale test. The vehicle condition adheres to the relevant provisions and collision criteria set forth in the standards, ensuring that the vehicle assembly is complete, has not exceeded its service life, and that the body parts and loading meet the technical requirements for normal operation. Prior to the test, the total mass, center of gravity position, and other technical indicators of the test vehicle are meticulously checked and recorded. The vehicle is then accelerated to the specified driving speed using a drop hammer to initiate the collision process.

The central dividing strip is excavated according to the design specifications, followed by backfilling and compaction, achieving a compaction degree of 81% for small passenger cars, 79% for medium-sized passenger cars, and 81% for medium-sized trucks. Tree pits are excavated, and trees are planted with planting soil backfilled without compaction. Curbs are installed in the dividing strip as per the design requirements. The construction and acceptance of the foundation adhere to the "Actual Vehicle Test Quality Control Standards."

### 4.2. Experience result

**Fig 8:**
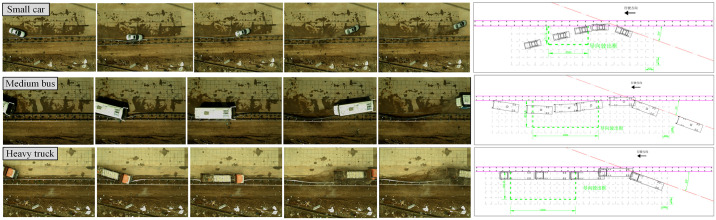
Driving trajectory of vehicle impacting guardrail.

Fig 8 shows the driving trajectory captured by a high-speed camera. It can be found that the vehicle’s driving trajectory after the collision meets the requirements of the guided exit frame.

**Fig 9:**
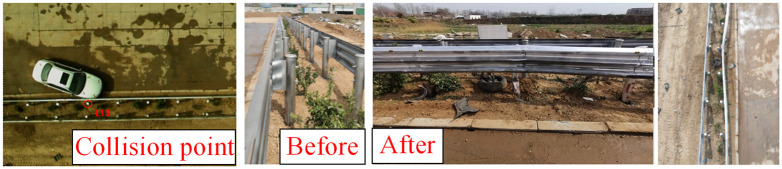
Deformation of guardrail after collision of small car.

Fig 9 presents an overall diagram of the full-scale collision test involving a small passenger car. The collision point is located at one-third of the length of the guardrail, specifically between columns No. 13 and No. 14 (closer to column No. 13). The entire collision process resulted in significant lateral displacement of columns No. 12 through No. 16, affecting a length of 10 meters of guardrail. The vehicle’s collision speed was recorded at 104 km/h, while the disengagement speed was 64 km/h, indicating that the collision resulted in a 62% loss of the vehicle’s kinetic energy.

The occupant risk assessment employs the Flail Space Model to evaluate the buffering function of the guardrail. The movement of the occupant’s head is categorized into two stages. In the first stage, following the collision with the highway guardrail, the vehicle decelerates while the hypothetical occupant’s head continues to move forward due to inertia, creating relative speed and displacement with the passenger compartment until it impacts the interior. In the second stage, after the head collides with the interior of the passenger compartment, its movement—specifically speed and acceleration—becomes synchronized with that of the vehicle.

As shown in Table 5, it takes 0.269 seconds for the hypothetical occupant’s head to travel 0.6 meters along the longitudinal axis (X direction) of the passenger compartment, and 0.245 seconds to move 0.3 meters along the horizontal axis (Y direction). The head collides with the interior of the passenger compartment at 0.245 seconds, resulting in occupant collision speeds of *V*_*x*_ = 3.6 m/s and *V*_*y*_ = 3.1 m/s, both of which are below the standard threshold of 12 m/s. The occupant collision accelerations are *a*_*x*_ = 8.8 m/s^2^ and *a*_*y*_ = 15.8 m/s^2^, both of which fall below the standard limit of 200 m/s^2^. Thus, the buffering function of the guardrail satisfies the established safety requirements.

**Table 5:** Buffering performance of small car.

Buffering index	Direction	Measure value	Standard requirement
OIV(m/s)	Axis X	3.6	≤12
	Axis Y	3.1	≤12
ORA(m/s^2^)	Axis X	8.8	≤200
	Axis Y	15.8	≤200

**Fig 10:**
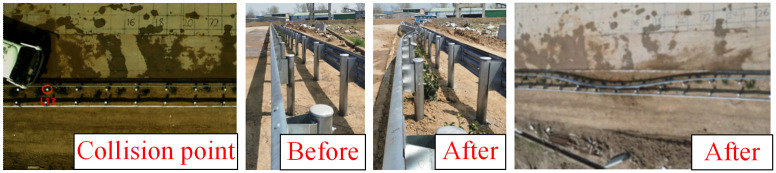
Deformation of guardrail after collision of medium bus.

Fig 10 presents an overall diagram of the full-scale collision test involving a medium-sized passenger car. The collision point is situated at one-third of the length of the guardrail, resulting in significant lateral displacement of columns No. 11 through No. 19. The length of the affected guardrail measures 16 meters. Notably, the vehicle did not penetrate, climb over, or straddle the guardrail, and no components of the guardrail were dislodged. The blocking function of the guardrail met the required safety standards. The vehicle’s collision speed was recorded at 82 km/h, while the disengagement speed was 66.4 km/h, leading to a 34% loss of the vehicle’s kinetic energy.

**Fig 11:**
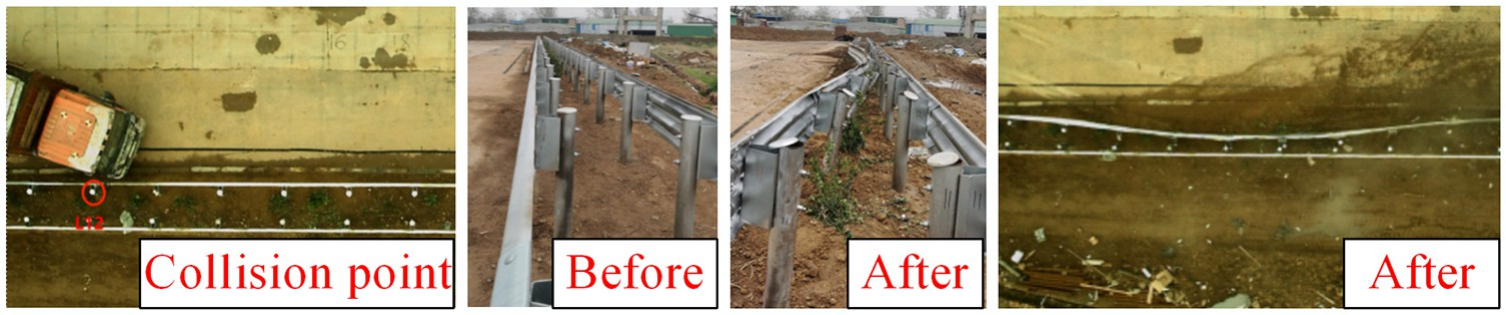
Deformation of guardrail after collision of heavy truck.

Fig 11 illustrates an overall diagram of the full-scale collision test involving a large truck. The collision point is located at one-third of the length of the guardrail, specifically between columns No. 12 and No. 13. The entire collision process resulted in significant lateral displacement of columns No. 10 through No. 20, affecting a length of 20 meters of guardrail. Importantly, the vehicle did not penetrate, climb over, or ride over the guardrail, and no components of the guardrail were dislodged. The blocking function of the guardrail met the required safety standards. The vehicle’s collision speed was recorded at 62 km/h, while the disengagement speed was 31.1 km/h, resulting in a 75% loss of the vehicle’s kinetic energy.

Table 6 presents the guardrail deformation and vehicle camber values. Full-scale tests conducted on three types of vehicles at specified collision angles and speeds (SB level) revealed that no vehicles penetrated, climbed over, or rode over the guardrails. Additionally, the test guardrail components and any detached parts did not encroach upon the vehicle passenger compartment, thereby fulfilling the blocking function requirements. The wheel track after exiting the departure point complied with the technical specifications of the standard guide exit frame, and the guardrail’s guidance function met the established standards. Furthermore, the buffer indicators for small passenger cars satisfied the required standards.

**Table 6:** Deformation of guardrail and vehicle camber value.

Metric	Small car	Medium bus	Heavy truck
Maximum dynamic lateral deflection of highway guardrai (D)	74.2	119.7	85
Maximum dynamic widening distance of lateral deflection of highway guardrail (W)	112.9	173	160.5
Incline-out angle (°)	-	12.5	18.0
Maximum dynamic vehicle incline-out distance (VI)	-	172.3	170.3
Normalized maximum dynamic vehicle incline-out distance (VI_n_)	-	190.9	189.5

## 5. Discussion and conclusions

This study proposes a high-strength, lightweight highway guardrail design. Through LS-DYNA finite element simulations and full-scale collision tests, all guardrail combinations effectively intercept moving vehicles without experiencing stumbling or deviation from the guide frame. The findings confirm that the various indicators of the SB-level new high-strength and lightweight guardrail fully meet the protective requirements stipulated for the corresponding level of guardrails in the specifications.

Taking China as a case study, it is estimated that highways and ordinary roads require approximately 5 million tons of highway corrugated beam steel guardrail products annually. Compared to conventional guardrails, high-strength steel lightweight corrugated beam guardrails can reduce steel consumption per kilometer by 44% (approximately 27.69 tons per kilometer), resulting in an annual steel savings of 2.2 million tons. Based on the calculation that each ton of steel generates 2.1 tons of CO_2_ emissions, this could lead to a reduction of 4.62 million tons of carbon emissions per year. Furthermore, with an estimated consumption of 571 kg of standard coal per ton of steel, this translates to a savings of 1.2562 million tons of standard coal annually.

Using an example of a transport vehicle with a capacity of 32 tons, traveling a distance of 1,000 km, the reduction in steel consumption could eliminate the need for approximately 68,750 transport vehicles, potentially decreasing vehicle transportation costs by about 44%. Assuming a diesel consumption of 0.0606 L/(t·km) for diesel trucks, and a carbon emission of 0.1553 kg/(t·km) [20], the total carbon dioxide emissions from transportation are estimated at around 342,000 tons, significantly conserving non-renewable resources such as coal and reducing overall energy consumption.

The new high-strength and lightweight guardrail demonstrates marked improvements in safety performance, along with lower construction and maintenance costs throughout its life cycle, presenting significant carbon reduction benefits and favorable conditions for industrialization and wider application. However, the standard section of the guardrail design examined in this study has not been further investigated concerning variations in guardrail height, curvature, and barrier block types. Additionally, parameters such as the relative position of the initial collision point, collision speed, and vehicle weight have utilized conventional values. Future studies may explore more extreme collision conditions to further enhance understanding.

## Supporting information

S1 File Collision test and index calculate. (DOCX)

S2 File SBm guradrail acceleration record (X axis). (XLS)

S3 File SBm guradrail acceleration record (Y axis). (XLS)

## References

[1] Li Z., Fang H., Fatoki J., Gutowski M., & Wang Q. (2021). A numerical study of strong-post double-faced W-beam and Thrie-beam guardrails under impacts of vehicles of multiple size classes. *Accident Analysis & Prevention*, 159, 106286. doi: 10.1016/j.aap.2021.106286 34271322

[2] Yao J., Wang B., Hou Y., & Huang L. (2021). Analysis of vehicle collision on an assembled anti-collision guardrail. *Sensors*, 21(15), 5152. doi: 10.3390/s21155152 34372389 PMC8348905

[3] Jaber M. M., Ali M. H., Abd S. K., Abosinnee A. S., & Malik R. Q. (2023). Simulation research on the collision between freight cars and expressway three-wave beam steel guardrail. *Multimedia Tools and Applications*, 1–22.

[4] LIU, Y., WU, Y., & LUO, S. (2008). LUO SuxingEffects of SO_4~(2-) Ions on Corrosion Behavior of Q235 Steel in Soil. *Journal of Southwest University*. *Natural Science Edition*,1673-9868(2008)30:7.

[5] Scouse A. A., Kelley S. S., Venditti R. A., & McConnell T. E. (2020). Evaluating sustainable product alternatives by combining life cycle assessment with full-cost accounting: a highway guardrail case study. *Bioresources*, 15(4), 9103.

[6] Zheng Y., Liao H., & Yang X. (2016). Stochastic pricing and order model with transportation mode selection for low-carbon retailers. *Sustainability*, 8(1): 48.

[7] Li, Y., & Zhang, Q. (2020, February). Research on carbon emission reduction based on the optimization of transportation structure under VAR model. In IOP Conference Series: *Earth and Environmental Science* (Vol. 440, No. 4, p. 042008). IOP Publishing.

[8] Yin H., Xiao Y., Wen G., & Fang H. (2017). Design optimization of a new W-beam guardrail for enhanced highway safety performance. *Advances in engineering software*, 112, 154–164.

[9] Jiang L., Wang K., Fang H., Chen B., Zhu L., Zhang Q., et al. (2024). Protection performance of a novel anti-collision guardrail with recycled foamed concrete under vehicle collision. *Engineering Structures*, 305, 117795.

[10] Yang J., Xu G., Cai C. S., & Kareem A. (2019). Crash performance evaluation of a new movable median guardrail on highways. *Engineering Structures*, 182, 459–472.

[11] Zhao K., Cui N., Zhao D., Ren D., & Shen X. (2021). Mechanism and prevention measures of post stumbling obstruction in car, guardrail collision. *Automotive Engineering*, 43(5), 746–753.

[12] Wang L., Huang X., Li R., Tang Z., Li J., & Chen D. (2024). Collision Study on New Aluminum Alloy W-Beam Guardrail. *Applied Sciences*, 14(12), 5266.

[13] Zhang A., Bu Q., Zhang W., He G., & Deng Y. (2024). Test and application of movable steel barrier with grade SB light composite corrugated beam. *Journal of Measurements in Engineering*, 12(1), 1–22.

[14] Li J., Wang Y., Ma Z., & Liang D. (2023). Simulation and performance analysis of passenger bus collision with rigid guardrail of expressway bridge. Bridge Structures, 19(1–2), 41–55.

[15] San Ha N., & Lu G. (2020). Thin-walled corrugated structures: A review of crashworthiness designs and energy absorption characteristics. *Thin-Walled Structures*, 157, 106995.

[16] Shakil S., Lu W., & Puttonen J. (2020). Experimental studies on mechanical properties of S700 MC steel at elevated temperatures. *Fire Safety Journal*, 116, 103157.

[17] Vuorinen E., Hosseini N., Hedayati A., Kornacker E., Fernandez M. T., Sanz J., et al. (2020). Mechanical and microstructural evaluation of high performance steel (S700MC) for road restraint systems. *Engineering Failure Analysis*, 108, 104251.

[18] Fu Z., Wu Z., and Jing L. (2010). Research on W-beam Guardrail Height of Expressway Median. *Journal of Highway and Transportation Research and Development*. 27.09(2010):143–148.

[19] JTG B05-01-2013; Standard for Safety Performance Evaluation of Highway Barriers. *China Communications Press*: Beijing, China, 2013.

[20] Yang, S. Overland freight economic service radius study based on carbon emission cost. Master’s Thesis, Beijing Jiaotong University, Beijing, China, 2014.

