# Supplementary figures and images for: Structural design and safety performance of a novel high-strength steel lightweight guardrail
Source: PLoS One. 2025 Jan 24;20(1):e0317353. doi: 10.1371/journal.pone.0317353 (PMC11759979; doi:10.1371/journal.pone.0317353)

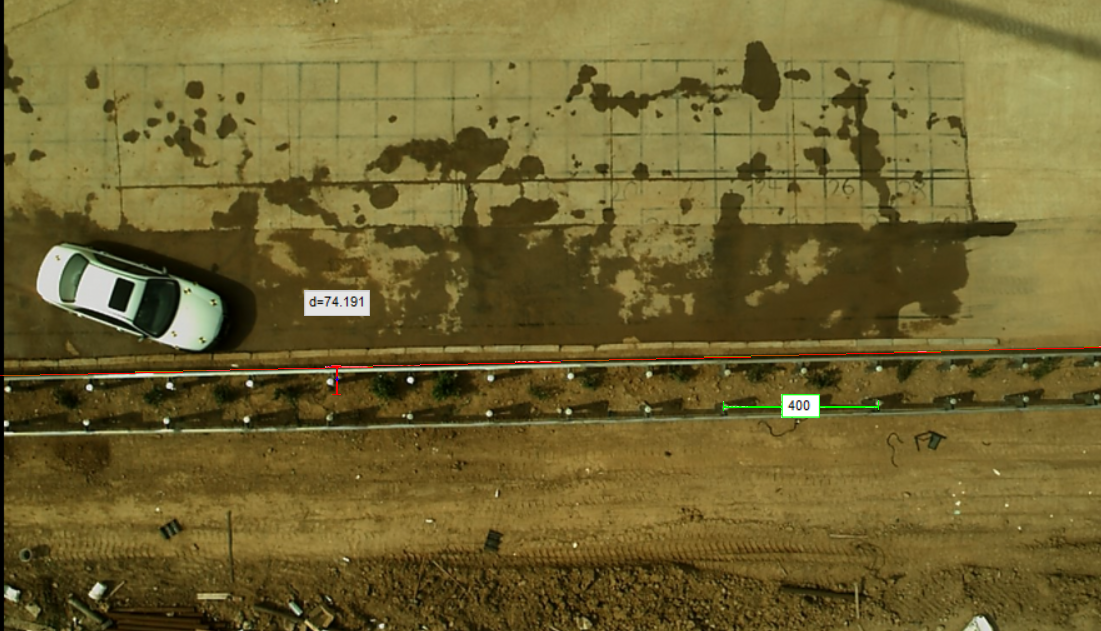

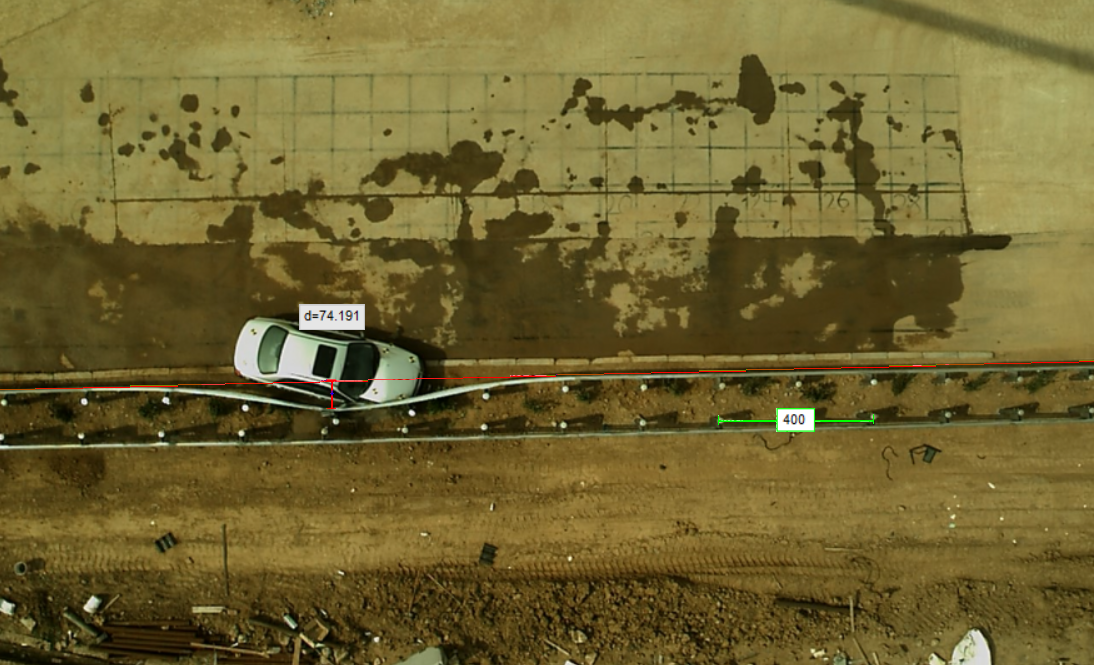
D：74


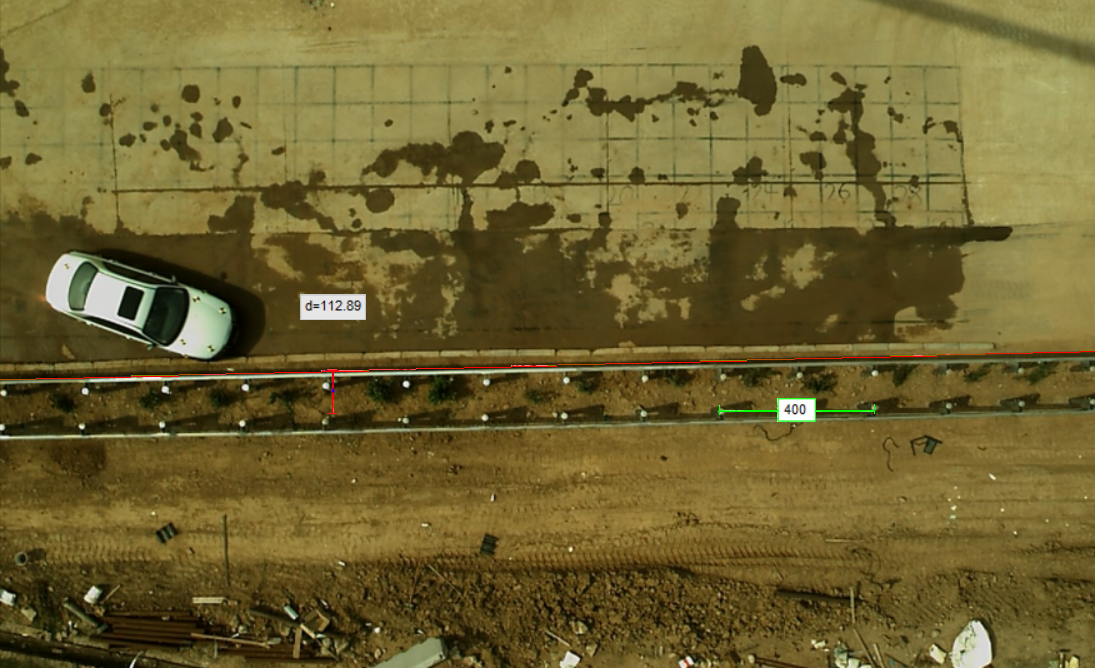

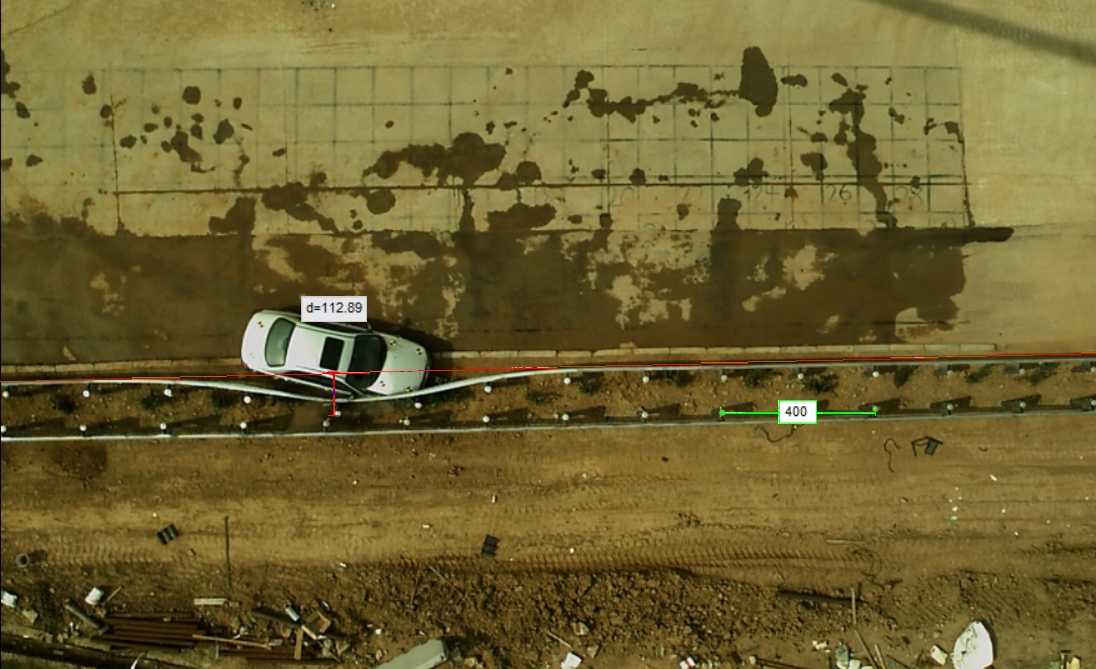
VI：113


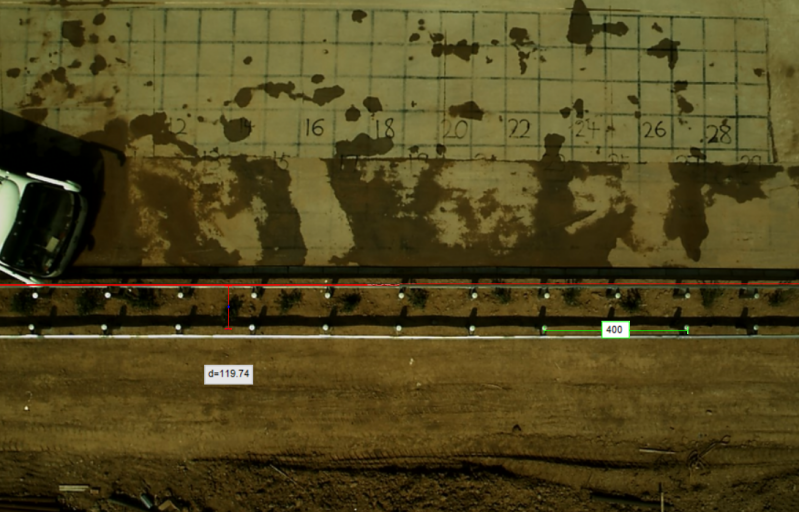

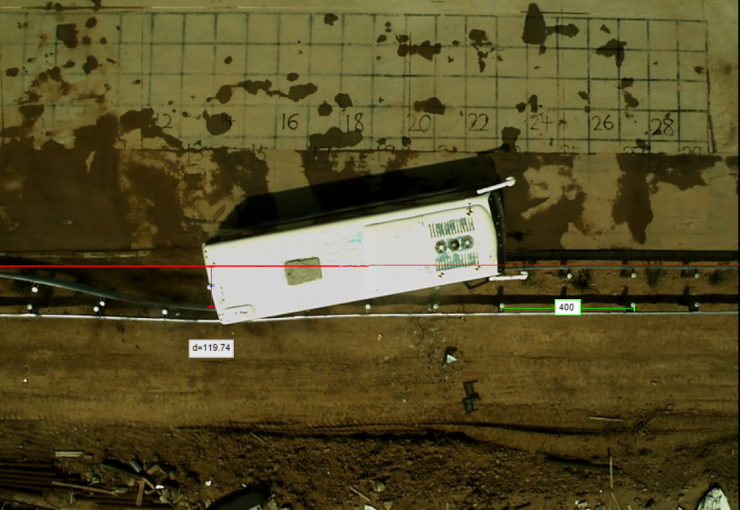
D：120


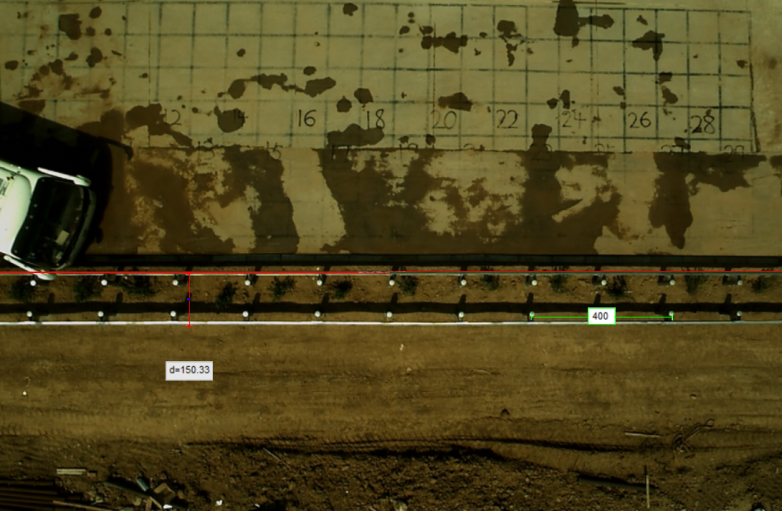

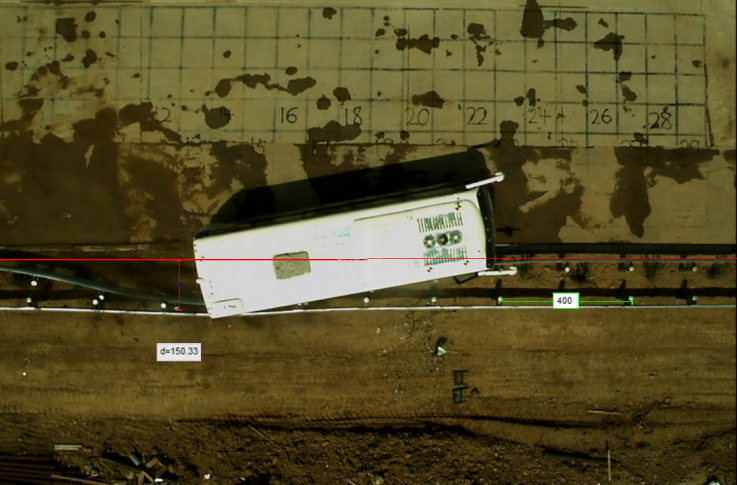
W：150


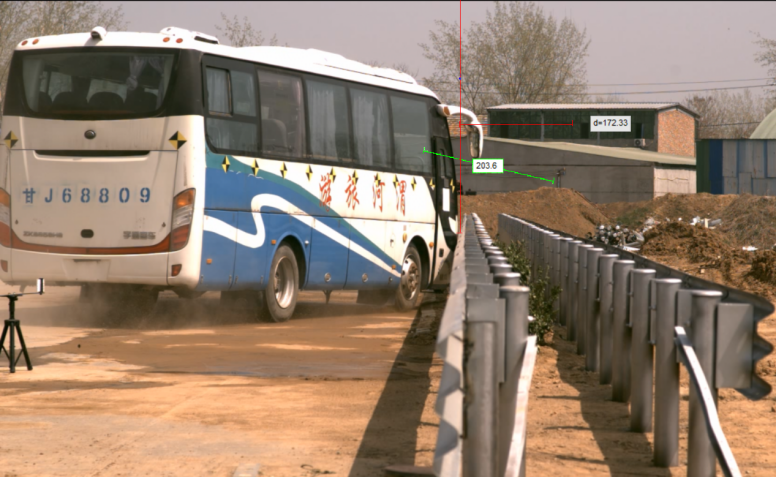

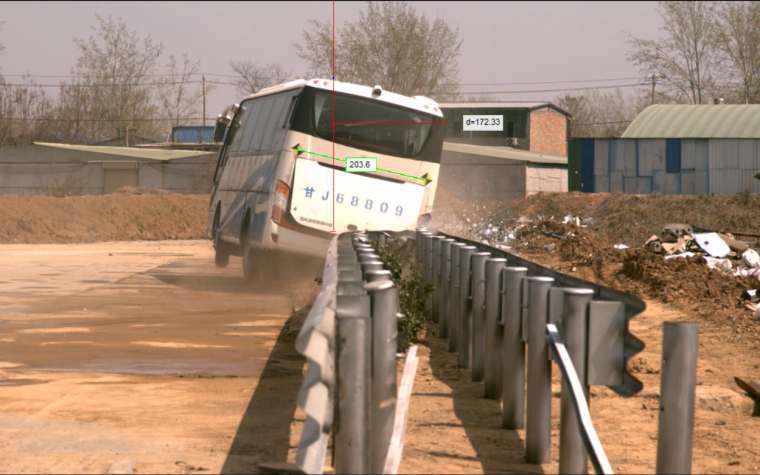
VI：172


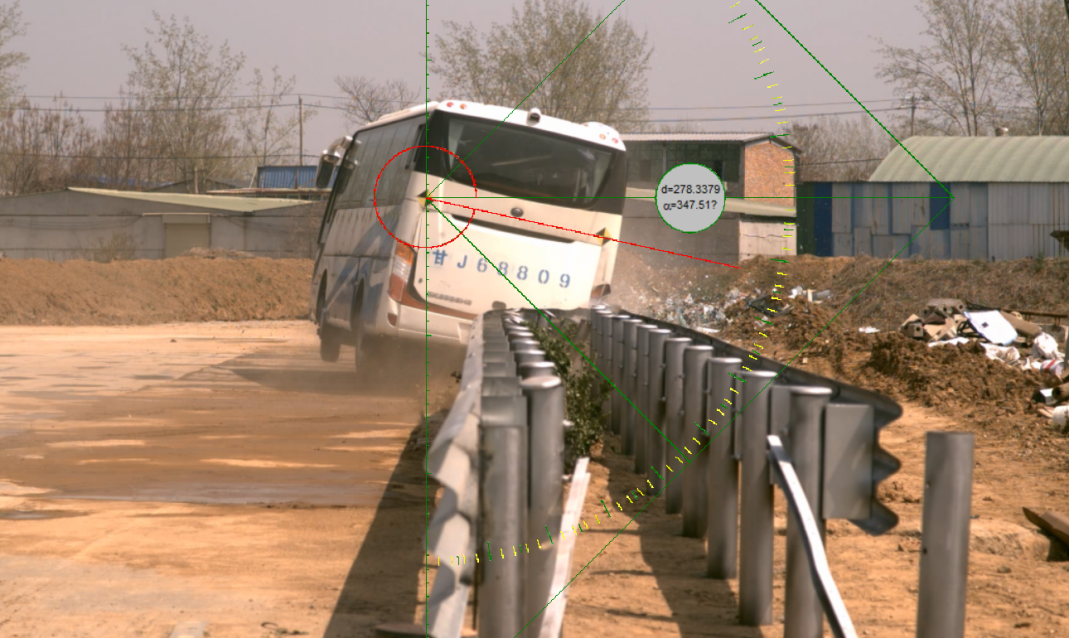


外倾角度α=12.49° VH=3.34m

VIn=1.72+（4.2-3.34）*

=1.90m


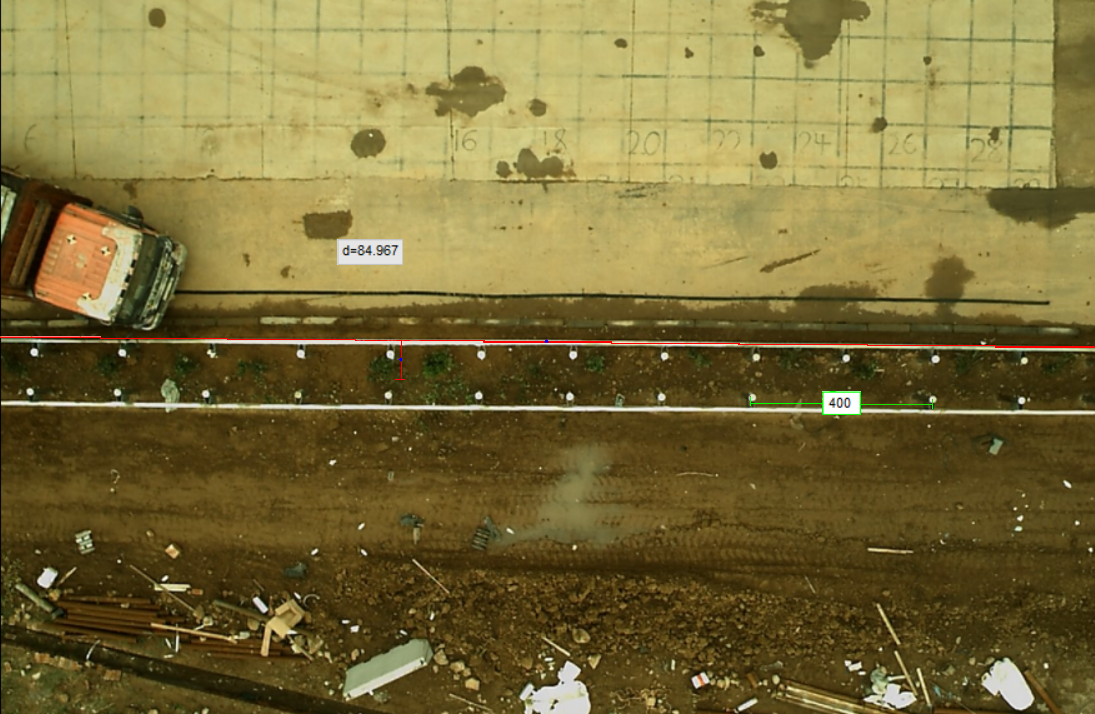

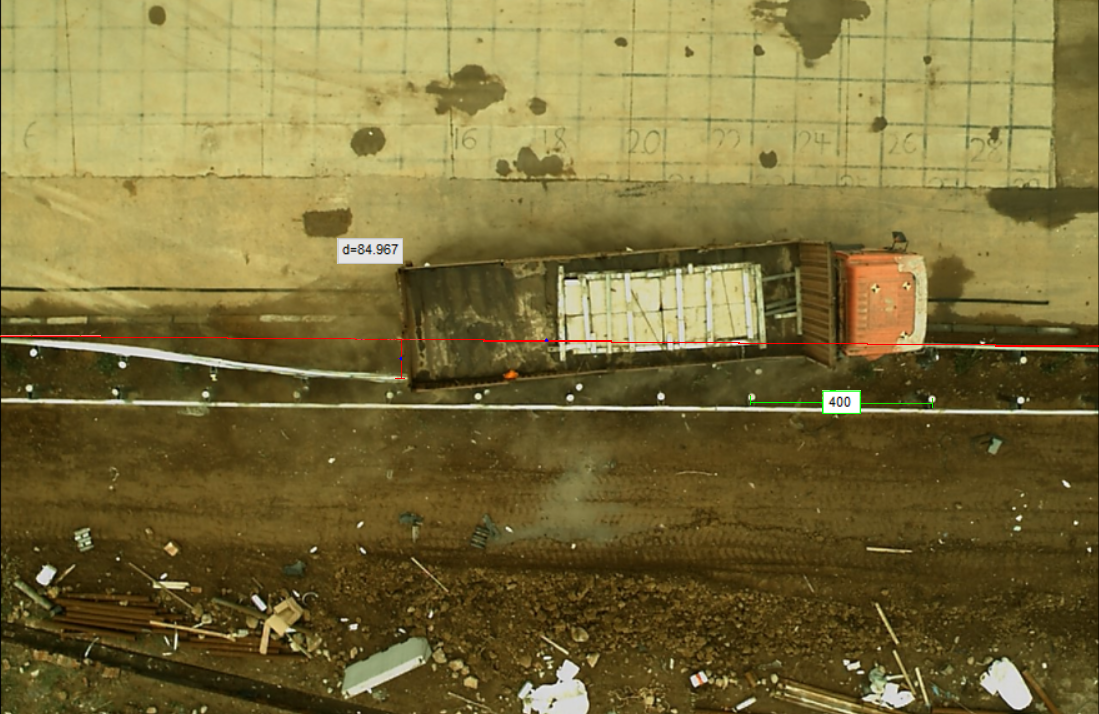
D：85


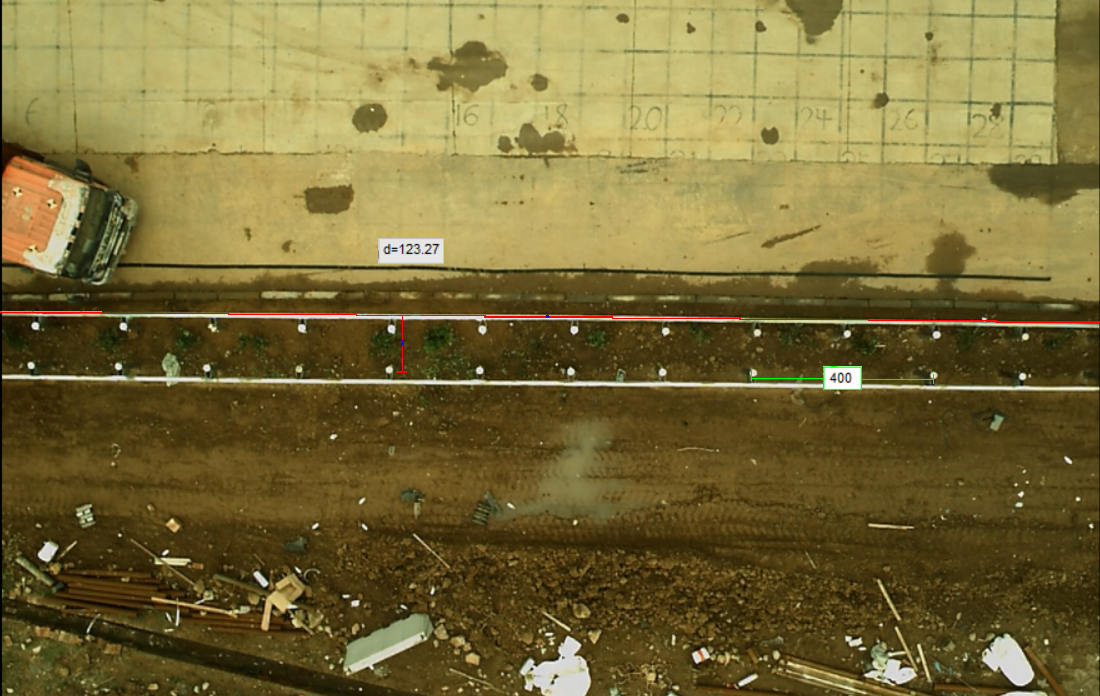

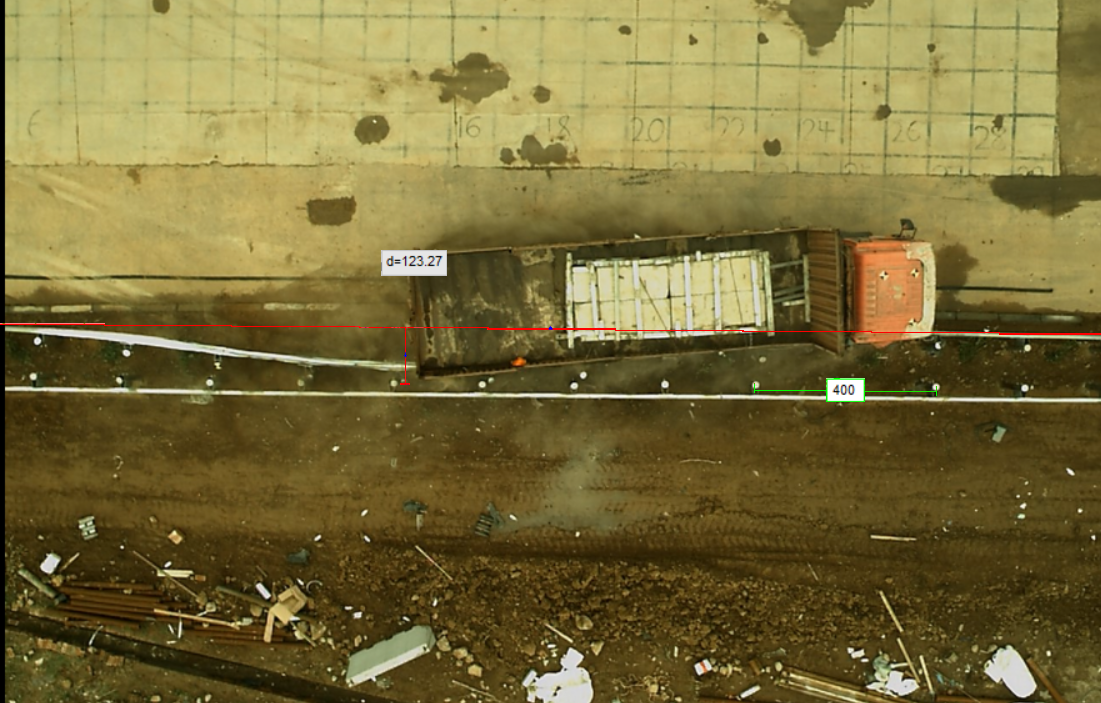
W:123


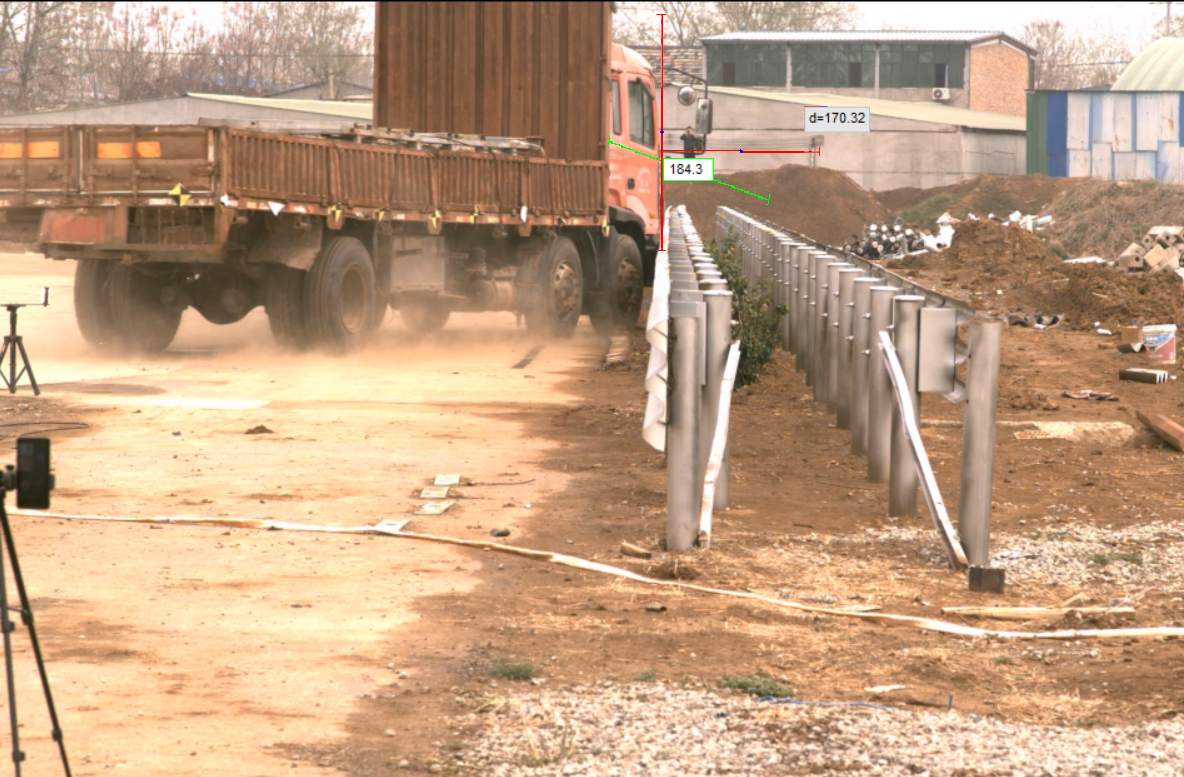

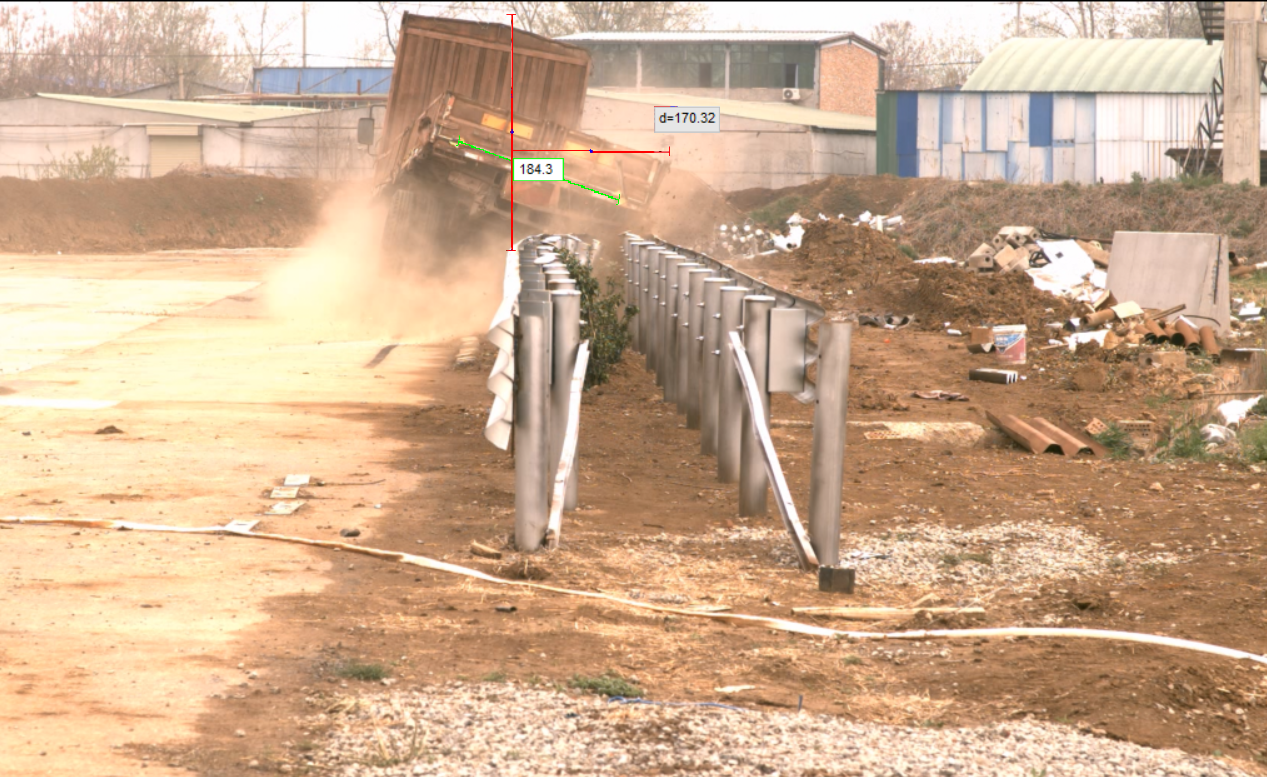
VI:170


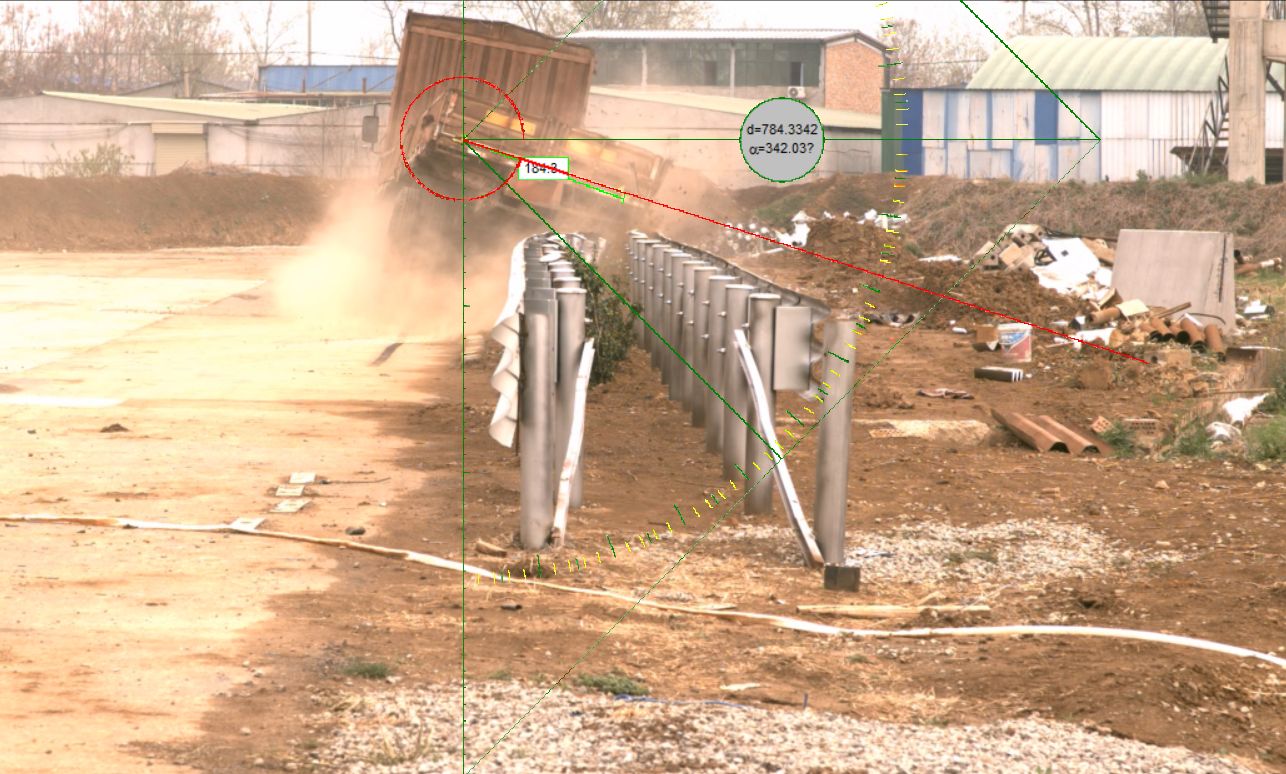


外倾角度α=17.97° VH=3.58m

VIn=1.7+（4.2-3.58）*

=1.89m


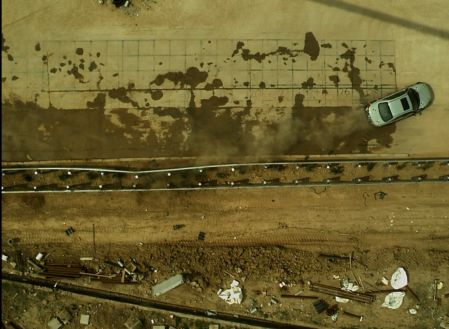

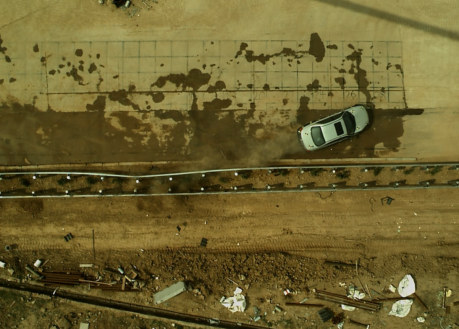

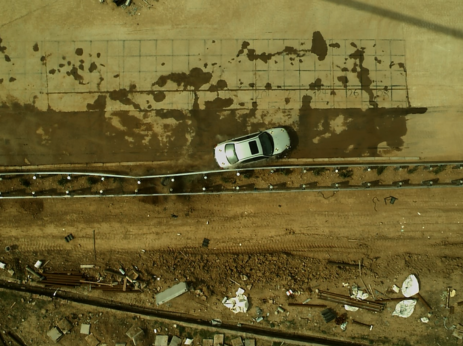

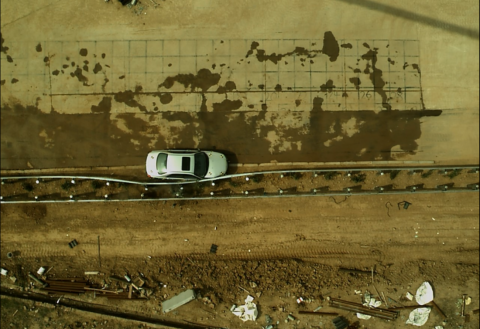

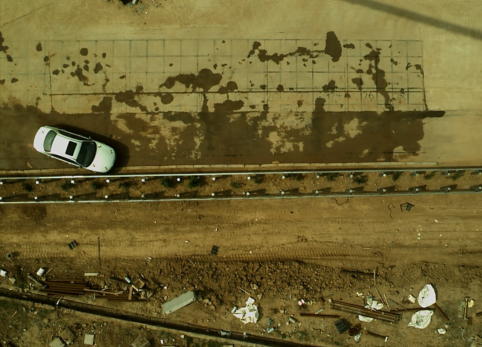


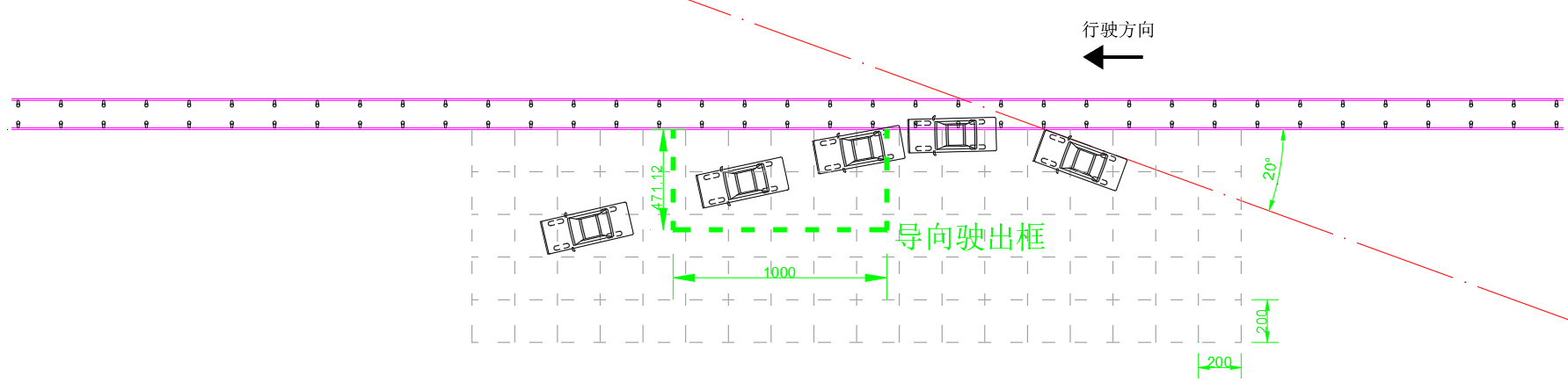


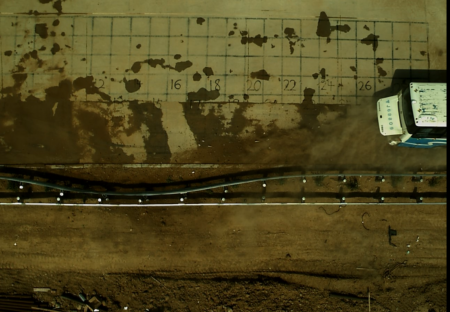

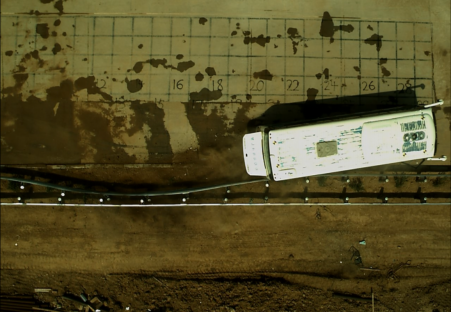

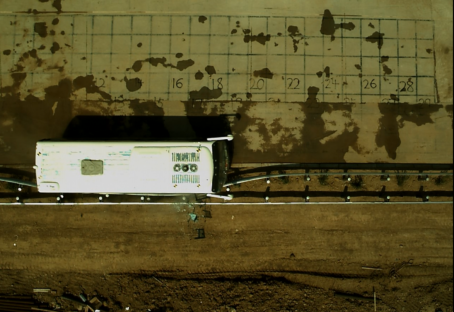

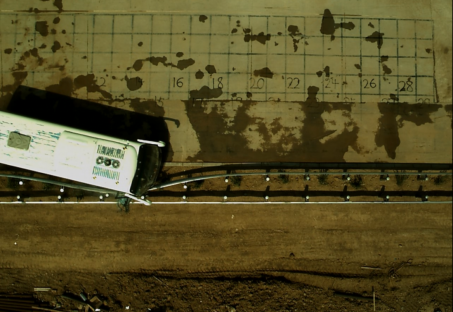

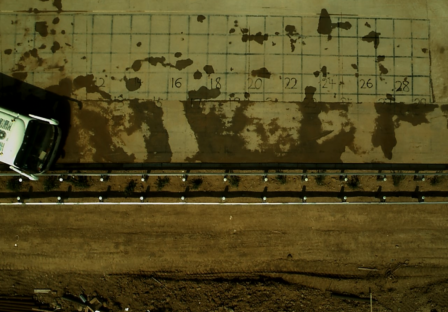


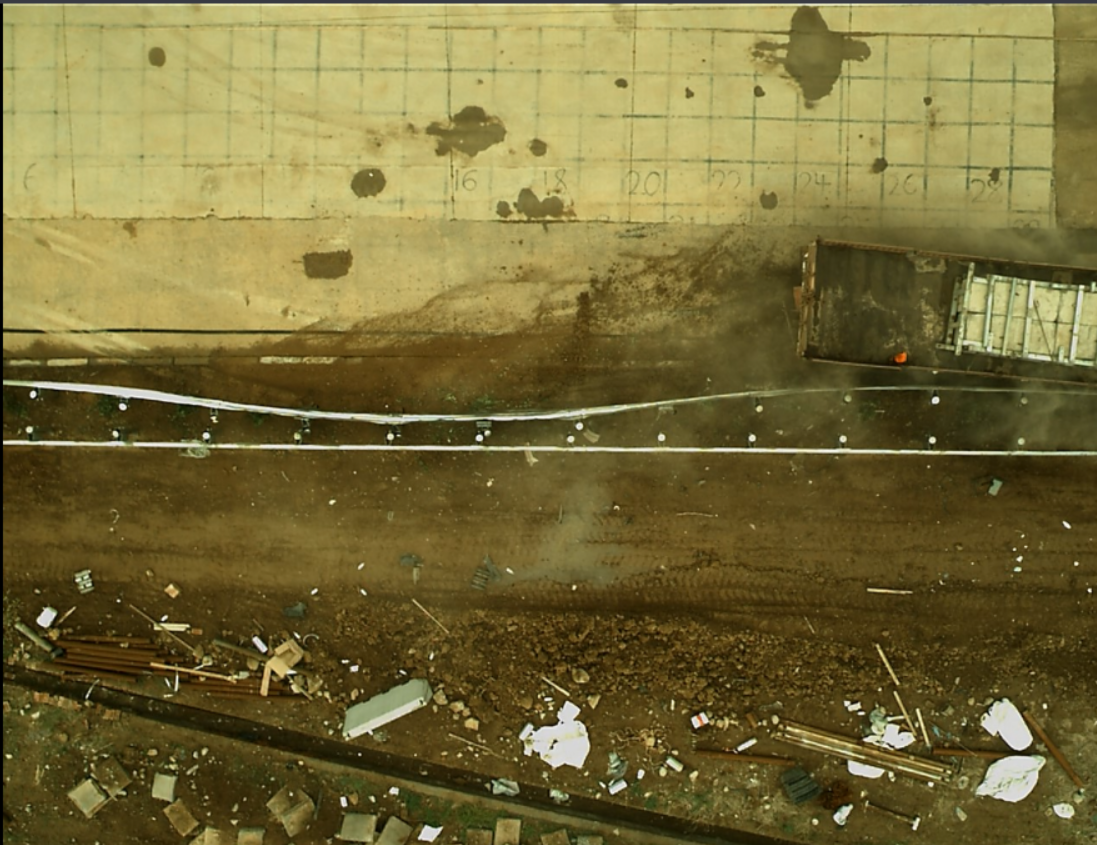

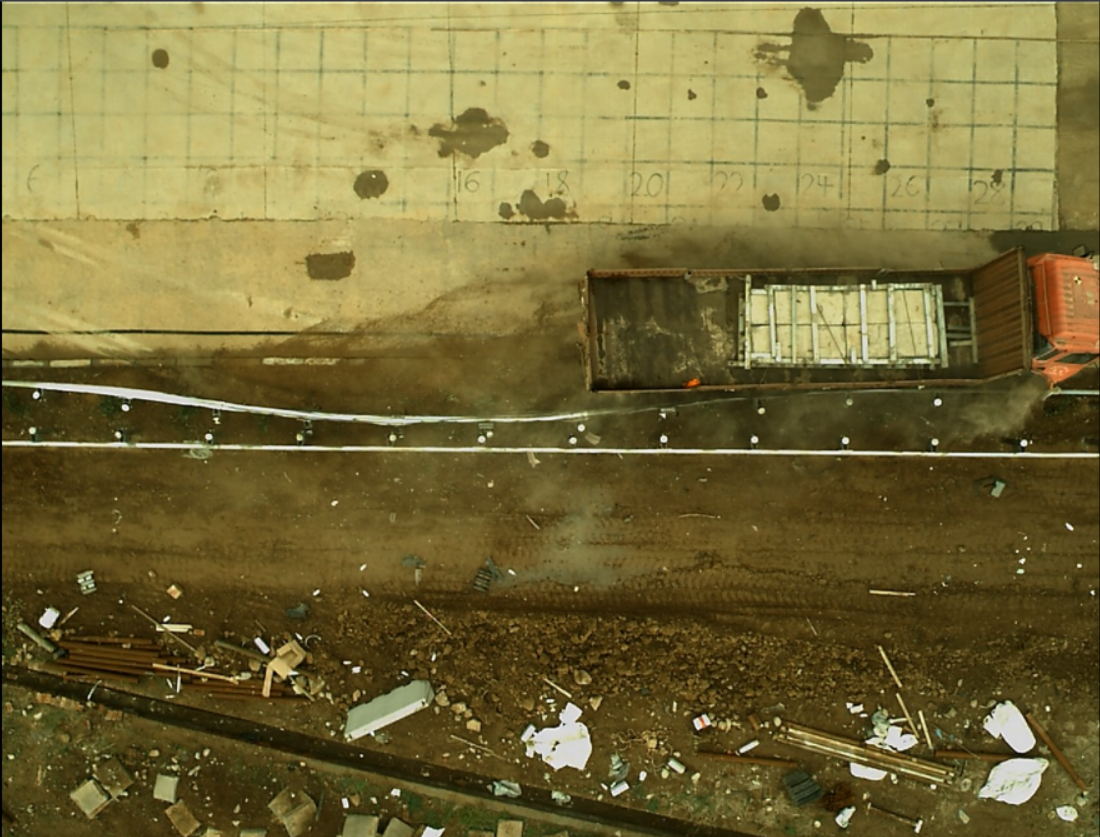

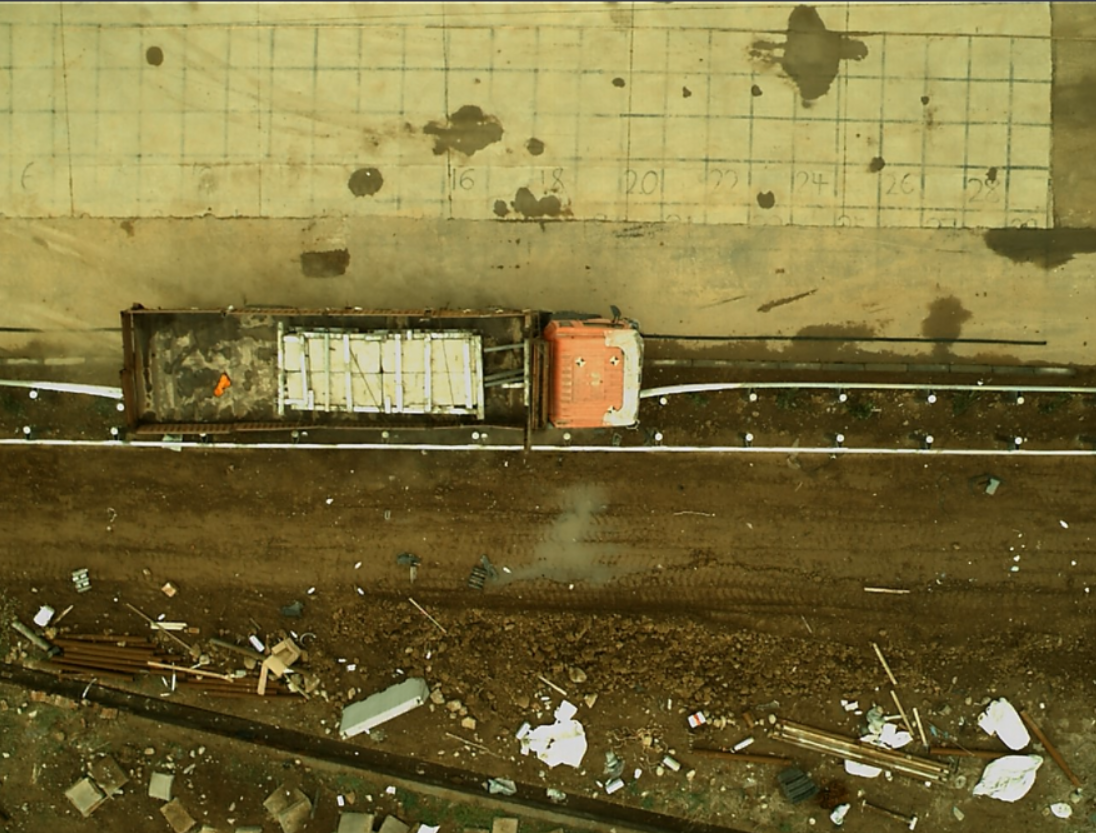

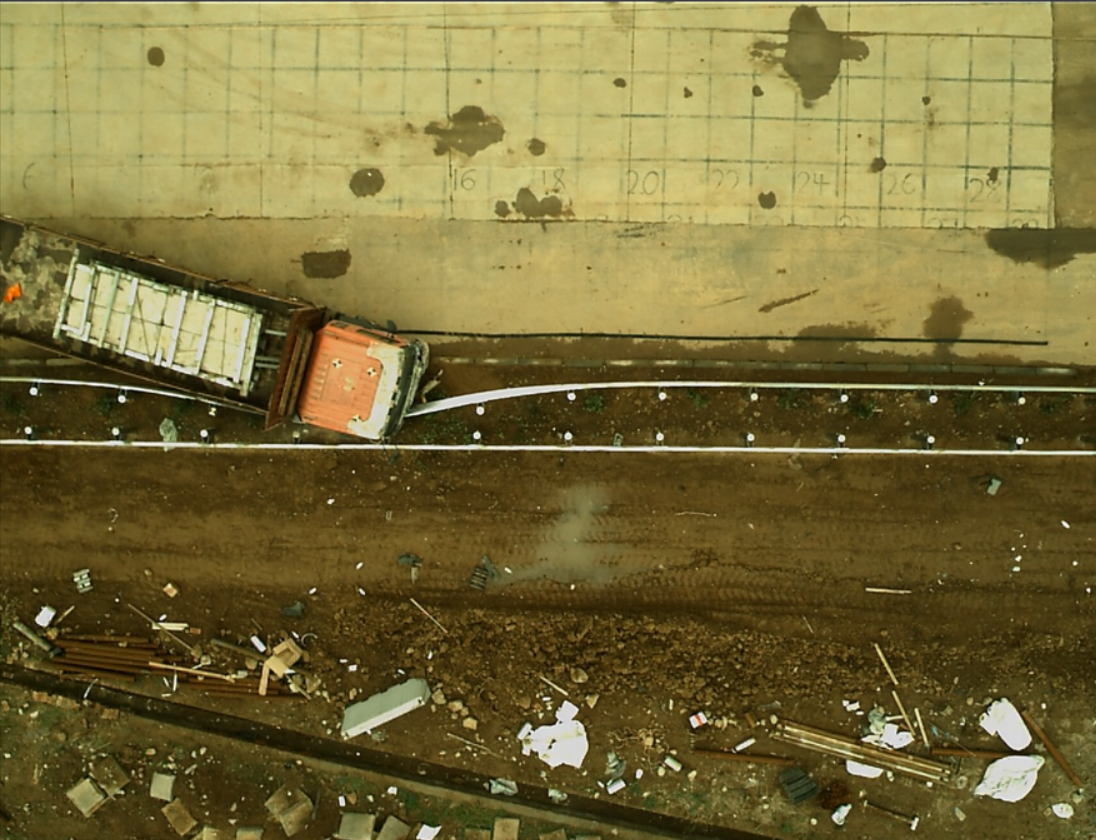

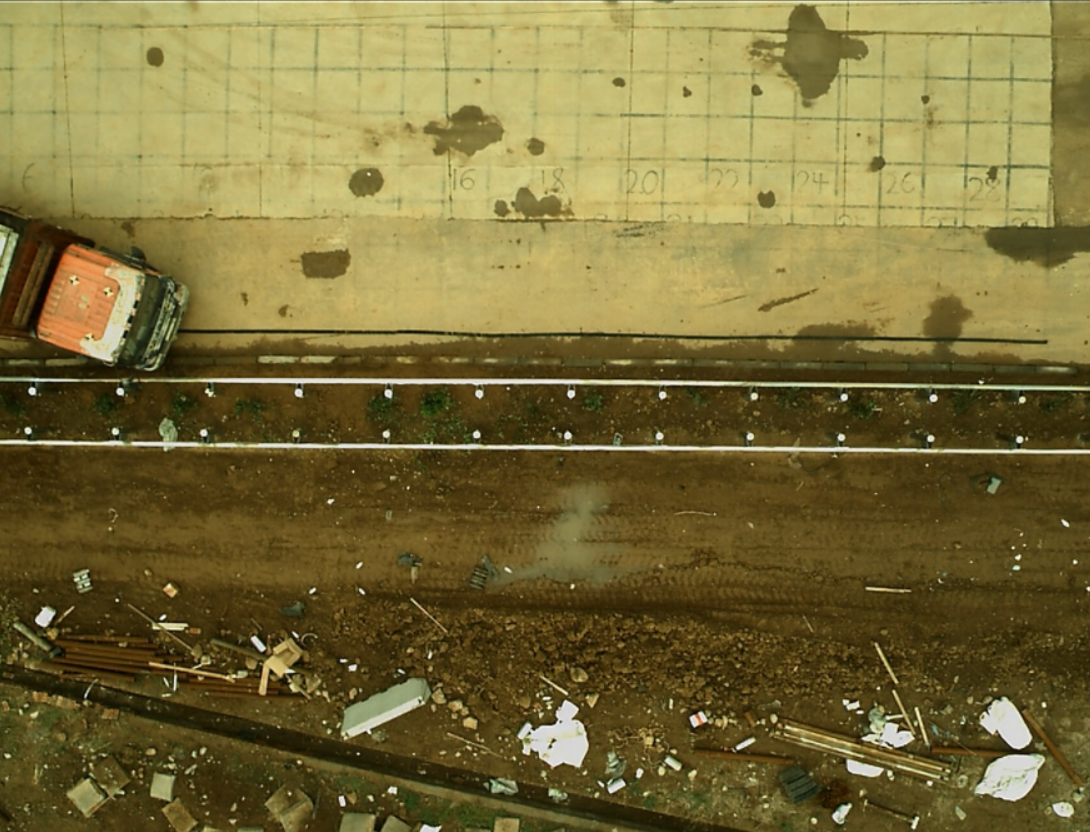


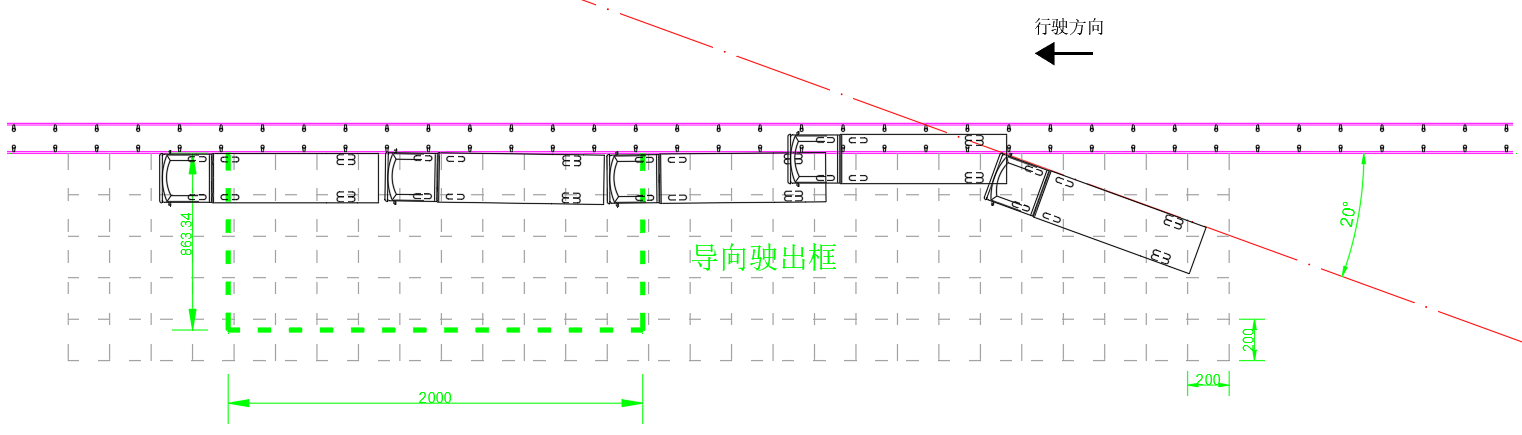

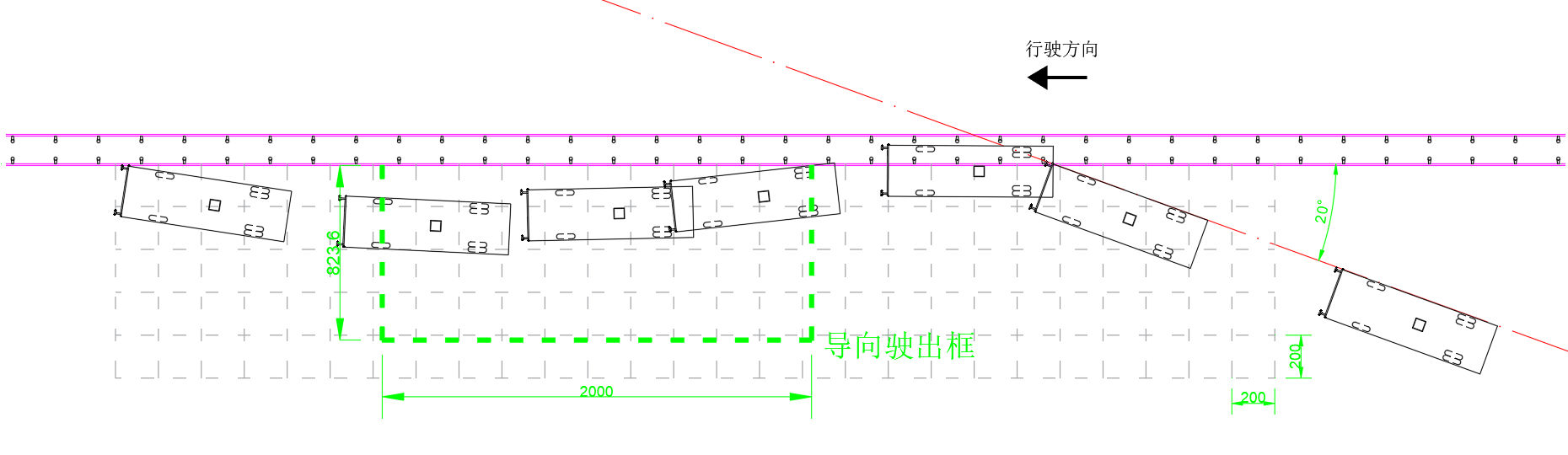

Supplement: S1 File — (DOCX) [file pone.0317353.s001.docx]
